# Development and Validation of an Electronic Health Record–Based Machine Learning Model to Estimate Delirium Risk in Newly Hospitalized Patients Without Known Cognitive Impairment

**DOI:** 10.1001/jamanetworkopen.2018.1018

**Published:** 2018-08-03

**Authors:** Andrew Wong, Albert T. Young, April S. Liang, Ralph Gonzales, Vanja C. Douglas, Dexter Hadley

**Affiliations:** 1 School of Medicine, University of California, San Francisco; 2 Clinical Innovation Center, Department of Medicine, University of California, San Francisco; 3 Department of Neurology, University of California, San Francisco; 4 Institute for Computational Health Sciences, University of California, San Francisco

## Abstract

**Importance:**

Current methods for identifying hospitalized patients at increased risk of delirium require nurse-administered questionnaires with moderate accuracy.

**Objective:**

To develop and validate a machine learning model that predicts incident delirium risk based on electronic health data available on admission.

**Design, Setting, and Participants:**

Retrospective cohort study evaluating 5 machine learning algorithms to predict delirium using 796 clinical variables identified by an expert panel as relevant to delirium prediction and consistently available in electronic health records within 24 hours of admission. The training set comprised 14 227 adult patients with non–intensive care unit hospital stays and no delirium on admission who were discharged between January 1, 2016, and August 31, 2017, from UCSF Health, a large academic health institution. The test set comprised 3996 patients with hospital stays who were discharged between August 1, 2017, and November 30, 2017.

**Exposures:**

Patient demographic characteristics, diagnoses, nursing records, laboratory results, and medications available in electronic health records during hospitalization.

**Main Outcomes and Measures:**

Delirium was defined as a positive Nursing Delirium Screening Scale or Confusion Assessment Method for the Intensive Care Unit score. Models were assessed using the area under the receiver operating characteristic curve (AUC) and compared against the 4-point scoring system AWOL (age >79 years, failure to spell *world* backward, disorientation to place, and higher nurse-rated illness severity), a validated delirium risk–assessment tool routinely administered in this cohort.

**Results:**

The training set included 14 227 patients (5113 [35.9%] aged >64 years; 7335 [51.6%] female; 687 [4.8%] with delirium), and the test set included 3996 patients (1491 [37.3%] aged >64 years; 1966 [49.2%] female; 191 [4.8%] with delirium). In total, the analysis included 18 223 hospital admissions (6604 [36.2%] aged >64 years; 9301 [51.0%] female; 878 [4.8%] with delirium). The AWOL system achieved a baseline AUC of 0.678. The gradient boosting machine model performed best, with an AUC of 0.855. Setting specificity at 90%, the model had a 59.7% (95% CI, 52.4%-66.7%) sensitivity, 23.1% (95% CI, 20.5%-25.9%) positive predictive value, 97.8% (95% CI, 97.4%-98.1%) negative predictive value, and a number needed to screen of 4.8. Penalized logistic regression and random forest models also performed well, with AUCs of 0.854 and 0.848, respectively.

**Conclusions and Relevance:**

Machine learning can be used to estimate hospital-acquired delirium risk using electronic health record data available within 24 hours of hospital admission. Such a model may allow more precise targeting of delirium prevention resources without increasing the burden on health care professionals.

## Key Points

**Question:**

Can machine learning be used to predict incident delirium in newly hospitalized patients using only data available in the electronic health record shortly after admission?

**Findings:**

In this cohort study, classification models were trained using 5 different machine learning algorithms on 14 227 hospital stays and validated on a prospective test set of 3996 hospital stays. The gradient boosting machine model performed best, with an area under the receiver operating characteristic curve of 0.855.

**Meaning:**

Machine learning can accurately predict delirium risk using electronic health record data on admission and outperforms the nurse-administered prediction rules currently used.

## Teaser

This study describes the development and prognostic accuracy of 5 machine learning models designed to predict incident delirium in newly hospitalized patients without known cognitive impairment using clinical variables derived from the patients’ electronic health record (EHR).

## Introduction

Delirium is common in hospitalized patients, with a prevalence of 18% to 35% and incidence of 11% to 14% in general medical wards, and is independently associated with poor health outcomes.^1^ It contributes between $38 billion and $152 billion per year to US health care costs.^2^ Current data suggest hospital-acquired incident delirium can be prevented in up to 53% of patients.^3^ Prevention strategies, however, are nonpharmacologic and therefore resource and personnel intensive.^4^ Accurate prediction of delirium risk could allow more precise targeting of high-risk patients and thereby greater resource stewardship and, potentially, improved patient outcomes.

Existing clinical delirium risk prediction tools have achieved areas under the receiver operating characteristic curve (AUCs) of 0.69 to 0.81.^5,6,7,8,9,10,11,12,13^ For example, UCSF Health (the University of California, San Francisco, Medical Center system) uses the AWOL screening tool to calculate delirium risk for newly admitted patients.^12^ This tool assigns 1 point for each of the following criteria: age greater than 79 years; inability to spell *world* backward; disorientation to city, state, county, hospital name, or floor; and nurse-rated moderate or severe illness severity. A score of 2 points or greater indicates high risk and helps direct hospital resources for delirium prevention (eg, rehabilitation services, patient care assistants, volunteers). A recent prospective cohort study at our institution found AWOL achieved an AUC of 0.73 on hospitalized patients aged 50 years or older.^13^

However, AWOL and other score-based delirium risk prediction tools often rely on questionnaires administered by health care professionals (eg, Mini-Mental State Examination), nonroutine clinical data (nursing subjective illness severity assessment), or additional calculations (eg, Acute Physiology and Chronic Health Evaluation score), making their integration into routine clinical workflow impractical. An external validation study of 4 such risk stratification tools describes the need to adapt and simplify prediction rules to allow use with routine clinical assessment data.^8^ Additionally, these tool development studies contain several limitations, including small sample size (N < 500), limitation of potential predictors to only those known a priori to be associated with delirium, and substantially lower performance on prospective validation compared with the retrospective cohort.

Furthermore, existing tools recapitulate well-studied delirium risk factors, such as cognitive impairment at baseline, delirium on admission, and severe illness.^5,6,7,8,9,10,11,12,13^ For this subpopulation of patients with unambiguous risk of developing hospital-acquired delirium, UCSF Health routinely provides delirium prevention precautions. However, it remains of crucial importance to identify and intervene on behalf of patients with elevated risk of incident delirium who lack these apparent risk factors on admission.

We developed and validated a machine learning model to predict hospital-acquired incident delirium in patients without baseline cognitive impairment, based only on data available in the electronic health record (EHR) within 24 hours of admission. To our knowledge, our data set of 18 223 hospitalization records represents the largest used to train and validate any delirium prediction model. Such an approach allows for (1) analysis of hundreds of clinical variables, (2) automated prediction without additional screening steps, thus reducing the burden on health care professionals, and (3) an application that may be readily integrated into the EHR for clinical decision support.

## Methods

### Ethical Review of Study and Waiver of Consent

The institutional review board at UCSF reviewed the protocol for this study and approved it as a quality improvement investigation. A waiver of written informed consent was granted by the UCSF institutional review board for this study. All data used in the study were deidentified prior to use.

### Study Population

**Figure 1.**
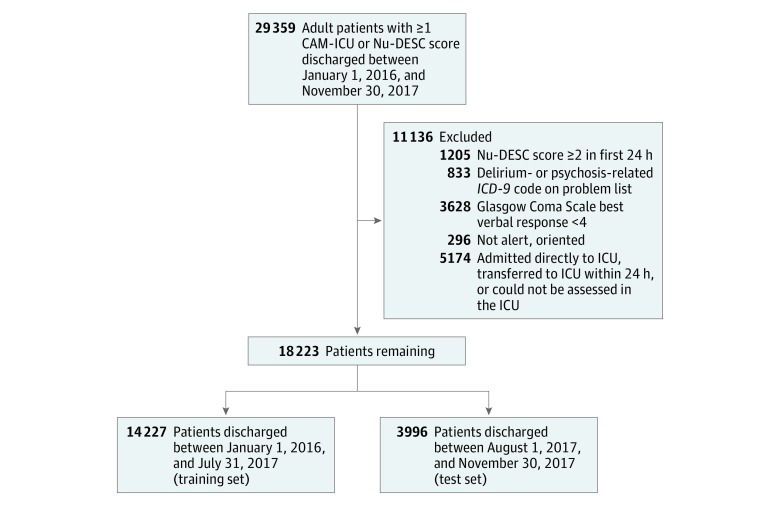
Study Flow Outlining Exclusion Criteria CAM-ICU indicates Confusion Assessment Method for the Intensive Care Unit; *ICD-9*, *International Classification of Diseases, Ninth Revision*; ICU, intensive care unit; Nu-DESC, Nursing Delirium Screening Scale.

Study data were collected retrospectively from UCSF Health’s EHRs. Unique hospitalizations, defined by contact serial numbers (CSNs), were included for adult patients discharged from UCSF Health between January 1, 2016, and November 30, 2017, and who had at least 1 Nursing Delirium Screening Scale (Nu-DESC) or Confusion Assessment Method for the Intensive Care Unit (CAM-ICU) screen performed within 30 days of admission. Inclusion and exclusion criteria are summarized in Figure 1. We excluded CSNs if patients were admitted with delirium, altered mental status, or illness severity requiring ICU admission, defined by 1 or more of the following: (1) a Nu-DESC score of 2 or greater within the first 24 hours; (2) an admission diagnosis or problem list including delirium, psychosis, or other alteration of consciousness (*International Classification of Diseases, Ninth Revision* [*ICD-9*] code 290.3, 290.11, 290.41, 291.0, 291.1, 292.81, 293.x, 295.x, 296.x, 297.x, 298.x, 300.11, 308, 780.09, or 780.39); (3) a Glasgow Coma Scale best verbal response score less than 4 on admission; (4) patient not alert and oriented to person, time, and place on admission; (5) patient admitted to the ICU, or transferred to the ICU within 24 hours after admission; and (6) patient spent time in the ICU and was unable to be assessed by CAM-ICU at any point. The first 5 criteria were chosen to exclude patients who were delirious on admission, those with obvious cognitive impairment, and patients receiving delirium interventions as part of routine care because of their presentation; the last criterion was chosen to avoid false-negatives in ICU patients.

The training set encompassed CSNs from discharges between January 1, 2016, and August 31, 2017; the test set comprised discharges between August 1, 2017, and November 30, 2017.

Race and ethnicity information was collected from the EHR patient demographics. Patients are asked to self-report their race and ethnicity at the time of hospital registration.

### Outcome Assessment

Nurses at UCSF Health collect Nu-DESC^14^ and CAM-ICU scores every 12 hours in medical-surgical units and the ICU, respectively, to screen for incident delirium.^15^ Incident delirium was defined as a Nu-DESC score of 2 or greater or a positive CAM-ICU result between 24 hours and 30 days after admission. We also performed a sensitivity analysis defining delirium as a Nu-DESC score of 1 or greater, which has a higher sensitivity for detecting delirium with a mild decrease in specificity.^16^

### Variable Selection

We compiled 796 clinical variables identified by an expert panel of health care professionals as relevant to delirium prediction and available in the EHR within 24 hours of admission, including admission diagnoses, medications, laboratory values, vital signs, and demographic and nursing data obtained during the admission assessment (eg, mobility, visual and hearing function, Glasgow Coma Scale, lines and tubes); microbiology, radiology, pathology, and procedures were not included (eTables 1 and 2 in the Supplement).

Apart from age, no AWOL criteria were included within our variable list. Only variables available within the first 24 hours of admission were considered to simulate timely prediction in the clinical setting. Admission diagnoses and problem lists were retrieved from the EHR in *ICD-9* format and were discretized into Boolean values for each of the 30 Elixhauser Comorbidity Index^17^ indicators using the R icd package (R Project for Statistical Computing). Home and admission medications were separately processed into Boolean values corresponding to 1 of 47 discrete categories based on the AHFS Pharmacologic-Therapeutic Classification,^18^ with the possibility of each medication being assigned multiple categories. For categorical variables, missing values were assigned to their own null category. For continuous variables, missing values were set to 0 and an indicator variable was added. The first value in alphabetical order for each categorical variable was chosen as the reference category, and the lowest value was chosen as the reference category for continuous variables.

### Model Training and Validation

We tested performance of 5 machine learning models in comparison to AWOL. Algorithms (R package implementation) comprised penalized logistic regression (glmnet), gradient boosting machine (gbm), artificial neural network with a single hidden layer (nnet), linear support vector machine (e1071), and random forest (randomForest). Using the R caret package,^19^ hyperparameters for each model were optimized with 3 repeats of 5-fold cross-validation, then fit to the entire training set. We then assessed each model by computing the AUC on the complete test set and the subset of hospitalizations in which an AWOL was performed. Model reporting complies with the Transparent Reporting of a Multivariable Prediction Model for Individual Prognosis or Diagnosis (TRIPOD) reporting guideline.^20^ Code and models have been made available at https://github.com/ayoung01/delirium.

### Statistical Analysis

We compared AUCs using a DeLong test for 2 correlated receiver operating characteristic (ROC) curves.^21^ A 2-sided level of significance of .05 was applied to general comparisons. All analyses were performed using R statistical software version 3.4.1 (R Project for Statistical Computing).

## Results

From 29 359 CSNs, we excluded 11 136 CSNs for delirium on admission or admission to the ICU (Figure 1). The rate of delirium in the cohort prior to application of the exclusion criteria was 13.5%. Of those excluded, 1205 CSNs (10.8%) had a Nu-DESC score of 2 or greater in the first 24 hours after admission. Among the remaining 9931 excluded CSNs (89.2%), the rate of incident delirium was 2909 of 9931 (29.3%) at a median (interquartile range [IQR]) of 2.3 (1.1-5.0) days after admission. Among included CSNs, the rate of incident delirium was 878 of 18 223 (4.8%) at a median (IQR) of 3.0 (1.8-5.7) days after admission, and the mean (SD) age was 57.1 (17.2) years. Of these 18 223 patients, 6604 (36.2%) were older than 64 years and 9301 (51.0%) were female. The training set comprised 14 227 adult patients with non-ICU hospital stays and no delirium on admission who were discharged between January 1, 2016, and August 31, 2017, from UCSF Health (5113 [35.9%] aged >64 years; 7335 [51.6%] female; 687 [4.8%] with delirium). The test set comprised 3996 patients with hospital stays who were discharged between August 1, 2017, and November 30, 2017 (1491 [37.3%] aged >64 years; 1966 [49.2%] female; 191 [4.8%] with delirium). Demographic characteristics did not differ meaningfully between the training and test sets (Table 1). The frequency of comorbidities was also similar between the 2 groups (eFigure 1 in the Supplement). eFigure 2 in the Supplement reports the number of included CSNs discharged each month by delirium outcome.

**Table 1.** Characteristics of the 18 223 Included Patients

Characteristic	No. (%)	
	Training Set (n = 14 227)	Test Set (n = 3996)
Age, y		
18-39	2589 (18.2)	743 (18.6)
40-64	6525 (45.9)	1762 (44.1)
65-79	3887 (27.3)	1118 (28.0)
>79	1226 (8.6)	373 (9.3)
Sex		
Male	6892 (48.4)	2030 (50.8)
Female	7335 (51.6)	1966 (49.2)
Race		
Asian	1751 (12.3)	514 (12.9)
Black	1443 (10.1)	391 (9.8)
Native Hawaiian or Pacific Islander	127 (0.9)	47 (1.2)
White	8372 (58.8)	2320 (58.1)
Other or declined	2534 (17.8)	724 (18.1)
Ethnicity		
Hispanic or Latino	1818 (12.8)	536 (13.4)
Not Hispanic or Latino	12 113 (85.1)	3391 (84.9)
Unknown or declined	296 (2.1)	69 (1.7)
Marital status		
Married	6620 (46.5)	1899 (47.5)
Single	5157 (36.2)	1447 (36.2)
Divorced or legally separated	1255 (8.8)	327 (8.2)
Widowed	994 (7.0)	255 (6.4)
Other or declined	201 (1.4)	68 (1.7)
Delirium^a^		
Yes	687 (4.8)	191 (4.8)
Age 18-64 y	330 (2.3)	86 (2.2)
Age >64 y	357 (2.5)	105 (2.6)
No	13 540 (95.2)	3805 (95.2)
Age 18-64 y	8784 (61.7)	2419 (60.5)
Age >64 y	4756 (33.4)	1386 (34.7)

^a^ Defined as Nursing Delirium Screening Scale score of 2 or greater or positive result for Confusion Assessment Method for the Intensive Care Unit at any time between 1 and 30 days after admission.

**Figure 2.**
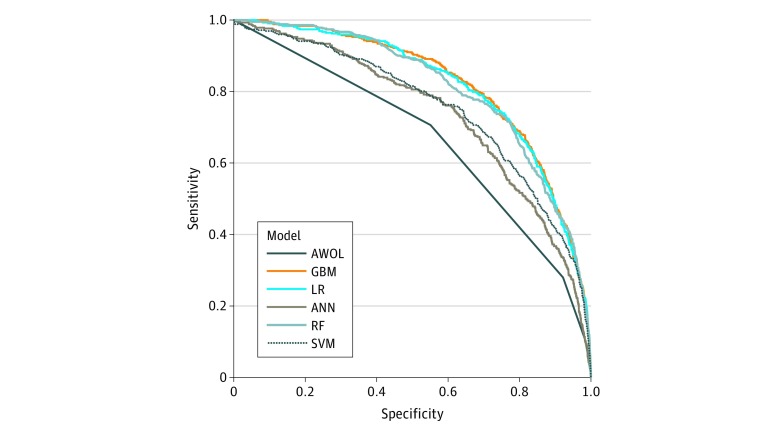
Receiver Operating Characteristic Curves for Machine Learning Models and AWOL Model performance was evaluated on a prospective test set (receiver operating characteristic curves shown are determined using the subset of the test set with AWOL [age, inability to spell *world* backward, orientation, illness severity] measurements). ANN indicates artificial neural network; GBM, gradient boosting machine; LR, penalized logistic regression; RF, random forest; and SVM, support vector machine.

Figure 2 summarizes the performance of each model. The AWOL system achieved an AUC of 0.678 with a sensitivity of 32.8% and a specificity of 90.5% at AWOL of 2 or greater. Scores on AWOL of 3 or greater achieved sensitivities of 14.4% and 2.4% and specificities of 97.9% and 99.8%, respectively. Gradient boosting machine (GBM), penalized logistic regression (LR), and random forest (RF) models performed best, with AUCs of 0.855, 0.854, and 0.848, respectively, on the complete test set, with no statistically significant difference between AUCs. The GBM, LR, and RF models achieved AUCs of 0.848, 0.845, and 0.843, respectively (*P* < .001 vs AWOL for each model), on the subset of the test set with an AWOL score within 24 hours of admission (n = 3356). eFigures 3 and 4 in the Supplement summarize the performance of these models stratified by age 18 to 64 years vs age greater than 64 years; our GBM model achieves an AUC of 0.856 and an AUC of 0.804 on these subgroups, respectively.

At the 90% specificity threshold, GBM achieved 59.7% (95% CI, 52.4%-66.7%) sensitivity, 90.0% (95% CI, 89.0%-90.9%) specificity, 23.1% (95% CI, 20.5%-25.9%) positive predictive value, 97.8% (95% CI, 97.4%-98.1%) negative predictive value, and a number needed to screen (NNS) of 4.8. Eighty-three of 191 cases of incident delirium (43.5%) were missed at this threshold. Forty-six of 114 true positives (40.4%) in patients younger than 65 years were correctly predicted at this threshold. At the 90% sensitivity threshold, GBM achieved 90.0% (95% CI, 84.9%-93.9%) sensitivity, 56.6% (95% CI, 55.0%-58.2%) specificity, 9.4% (95% CI, 8.9%-10.0%) positive predictive value, 99.1% (95% CI, 98.7%-99.4%) negative predictive value, and an NNS of 12. The confusion matrix metrics describing the performance of GBM, LR, and RF and AWOL of 2 or greater are reported in eTable 3 in the Supplement, and the corresponding confusion matrices are reported in eTables 4 to 10 in the Supplement.

From 796 initial variables, GBM selected 345 variables, LR selected 114, and RF selected 588. The 40 most predictive variables occurring in at least 10 samples from GBM are summarized in Table 2 and Table 3. In addition, we report whether these predictors were selected among the top 50 variables by LR and RF.

**Table 2.** Categorical Variables With Top Importance by Gradient Boosting Machine Occurring in at Least 10 Samples

Variable	Variable Category	Variable Importance^a^	Variable Frequency by Delirium Status, No. (%)		Selection by Other Models^c^
			Yes (n = 191)^b^	No (n = 3805)	
Neurologic examination					
Best verbal response	4	100.0	65 (34.0)	138 (3.6)	RF, LR
Neurologic symptoms (other)	Yes	13.2	25 (13.1)	116 (3.0)	RF, LR
Best motor response (upper extremities)	5	10.2	5 (2.6)	23 (0.6)	RF, LR
Best eye response	3	2.7	35 (18.3)	226 (5.9)	RF, LR
Best motor response (upper extremities)	6	1.1	104 (54.5)	1929 (50.7)	RF
Admission status					
Source	Transfer-acute hospital	17.8	38 (19.9)	237 (6.2)	RF, LR
Category	Urgent	4.8	65 (34.0)	684 (18.0)	RF, LR
Service	Neurology	2.6	13 (6.8)	166 (4.4)	RF, LR
Department	Neurosciences	1.7	20 (10.5)	246 (6.5)	RF, LR
Department	Other	1.6	132 (69.1)	2367 (62.2)	RF
Readmission (ie, recent hospitalization within prior 30 d)	Yes	1.3	27 (14.1)	507 (13.3)	LR
Activities of daily living					
Elimination	Incontinence	8.5	36 (18.8)	116 (3.0)	RF, LR
Feeding	Independent	6.8	114 (59.7)	3264 (85.8)	RF, LR
Bowel and bladder habits	Unable to assess	6.4	2 (1.0)	24 (0.6)	RF, LR
Grooming	Independent	4.0	73 (38.2)	2825 (74.2)	RF, LR
Bathing	Independent	1.1	59 (30.9)	2533 (66.6)	RF, LR
Home medications and devices					
Psychotherapeutic agents	Yes	5.7	83 (43.5)	1253 (32.9)	RF, LR
Parasympathomimetic or cholinergic agents	Yes	3.1	9 (4.7)	38 (1.0)	LR
Antimanic agents	Yes	2.3	3 (1.6)	37 (1.0)	NS
Devices	Yes	1.0	24 (12.6)	416 (10.9)	NS
Admission medications and devices					
Antimigraine agents	Yes	1.8	0	25 (0.7)	NS
Abdominal binder	Yes	1.6	11 (5.8)	96 (2.5)	NS
β-Adrenergic blocking agents	Yes	1.4	3 (1.6)	15 (0.4)	LR
Indwelling urinary Foley catheter	NA	1.4	108 (56.5)	2378 (62.5)	RF
Analgesic and antipyretics	Yes	1.4	37 (19.4)	631 (16.6)	LR
Diagnostic agents	Yes	1.2	0	19 (0.5)	NS
Opiate antagonists	Yes	1.0	9 (4.7)	166 (4.4)	NS
Comorbidities					
Depression	Yes	3.1	1	25 (0.7)	LR
Peripheral vascular disease	Yes	3.0	4 (2.1)	73 (1.9)	LR
Pulmonary disease	Yes	1.6	3 (1.6)	102 (2.7)	NS
Liver disease	Yes	1.6	9 (4.7)	99 (2.6)	LR
Alcohol use	Yes	1.6	4 (2.1)	33 (0.9)	LR
Difficulty chewing	Yes	1.5	12 (6.3)	172 (4.5)	NS
Nonhealing wound	NA	1.5	27 (14.1)	213 (5.6)	NS
Tumor	Yes	1.0	23 (12.0)	280 (7.4)	LR
Renal disease	Yes	1.0	12 (6.3)	104 (2.7)	NS
Mobility and fall risk					
Schmid fall score	4	1.9	1 (0)	18 (0.5)	NS
Mobility	Unable to ambulate or transfer	1.4	42 (22.0)	293 (7.7)	RF
Schmid fall score	3	1.1	20 (10.5)	150 (3.9)	LR
Patient demographic characteristics					
Race	Asian	1.9	25 (13.1)	489 (12.9)	LR

Abbreviations: LR, penalized logistic regression; NA, not applicable; NS, not selected; RF, random forest.

^a^ Rather than *P* values or coefficients, the gradient boosting machine model reports the importance of predictor variables included in a model. Importance is a measure of each variable’s cumulative contribution toward reducing square error, or heterogeneity within the subset, after the data set is sequentially split based on that variable. Thus, it is a reflection of a variable’s impact on the predictor. Absolute importance is then scaled to give relative importance, with a maximum importance of 100.

^b^ Defined as Nursing Delirium Screening Scale score of 2 or greater or positive result for Confusion Assessment Method for the Intensive Care Unit at any time between 1 and 30 days after admission.

^c^ Variable selected by model and ranked among top 50 in importance.

**Table 3.** Continuous Variables With Top Importance Selected by Gradient Boosting Machine and Coselection by Random Forest and Penalized Logistic Regression

Variable	Variable Importance^a^	Value by Delirium Status, Mean (SD)^b^		Selection by Other Models^d^
		Yes (n = 191)^c^	No (n = 3805)	
Patient demographic characteristics				
Age, y	18.6	65.0 (15.7)	57.0 (17.3)	RF, LR
Time since onset of pain, d	1.4	530 (1109)	560 (1790)	RF
Vitals				
Temperature, °F	17.0	97.1 (7.9)	97.5 (3.8)	RF
Heart rate, beats/min	8.3	88.4 (20.5)	78.7 (22.8)	RF, LR
Respiratory rate, breaths/min	7.5	12.7 (2.4)	13.4 (4.1)	RF, LR
NR average diastolic blood pressure, mm Hg	7.2	60.6 (11.7)	61.4 (10.4)	RF
NR average systolic blood pressure, mm Hg	6.6	104.6 (7.8)	106.4 (8.6)	RF, LR
Spo_2_, %	0.9	99.1 (2.7)	98.2 (4.6)	RF
Comprehensive metabolic panel				
Calcium, mg/dL	6.8	8.8 (0.8)	8.8 (0.7)	RF
Total bilirubin, mg/dL	5.4	1.5 (3.2)	1.3 (2.5)	RF, LR
Chloride, mmol/L	5.3	101.7 (6.2)	102.6 (5.3)	RF
Minimum BUN, mg/dL	4.5	28.1 (25.3)	19.7 (17.7)	RF, LR
AST, units/L	2.8	65.0 (148.9)	56.1 (170.2)	RF
Maximum glucose, mg/dL	2.1	137.4 (52.8)	138.0 (62.9)	RF, LR
Bicarbonate, mmol/L	2.0	25.0 (4.3)	24.7 (4.0)	RF
Ammonia, μmol/L	1.4	33.0 (NC)	41.4 (26.5)	RF
ALT, units/L	1.2	46.8 (95.9)	50.0 (169.4)	RF
CBC				
Platelet, ×10^3^/μL	6.5	240.2 (130.7)	238.1 (108.1)	RF
Hematocrit, %	2.5	34.4 (6.4)	35.3 (6.5)	RF

Abbreviations: ALT, alanine aminotransferase; AST, aspartate aminotransferase; BUN, blood urea nitrogen; CBC, complete blood cell count; LR, penalized logistic regression; NC, not calculable; NR, nursing record; RF, random forest; Spo_2_, oxygen saturation as measured by pulse oximetry.

SI conversion factors: To convert calcium to mmol/L, multiply by 0.25; AST and ALT to μkat/L, multiply by 0.0167; total bilirubin to μmol/L, multiply by 17.104; glucose to mmol/L, multiply by 0.0555; and platelet count to ×10^9^, multiply by 1.0.

^a^ Rather than *P* values or coefficients, the gradient boosting machine model reports the importance of predictor variables included in a model. Importance is a measure of each variable's cumulative contribution toward reducing square error, or heterogeneity within the subset, after the data set is sequentially split based on that variable. Thus, it is a reflection of a variable's impact on the predictor. Absolute importance is then scaled to give relative importance, with a maximum importance of 100.

^b^ Mean values are calculated excluding missing values.

^c^ Defined as Nursing Delirium Screening Scale score of 2 or greater or positive Confusion Assessment Method for the Intensive Care Unit result at any time between 1 and 30 days after admission.

^d^ Variable selected by model and ranked among top 50 in importance.

Using a more sensitive definition of delirium (replacing Nu-DESC score ≥2 with Nu-DESC score ≥1), AWOL achieved a baseline AUC of 0.666, and the AUCs for GBM, LR, RF, artificial neural networks (ANN), and support vector machine models achieved AUCs of 0.822, 0.820, 0.811, 0.736, and 0.759, respectively, on the complete test set (eFigure 5 in the Supplement). The *P* values for a DeLong test comparing ROC curves calculated using the definitions of Nu-DESC score greater than or equal to 1 and Nu-DESC score greater than or equal to 2 are .19, .19, .12, .44, and .046, for GBM, LR, RF, ANN, and support vector machine models, respectively.

We conducted a sensitivity analysis to test for bias introduced by patients with multiple hospitalizations by removing the 702 medical record numbers (19.9%) in the test set that overlapped with those of the training set, but performance of the GBM model was unaffected (AUC, 0.857).

## Discussion

This study demonstrates that machine learning models outperform current clinical tools used to assess delirium risk. In comparison with AWOL, which was found to have an NNS of 11.1 at the threshold of AWOL greater than or equal to 2, our GBM model achieves an NNS of 4.8 while maintaining a higher sensitivity than AWOL, suggesting that fewer than half as many patients would need to be treated for 1 to benefit from delirium prevention interventions. Machine learning models have the additional advantage of not requiring a health care professional to perform a bedside delirium risk assessment.

As with any diagnostic test, the choice of threshold for specificity or sensitivity depends on the interventions triggered by a positive screen. A high specificity threshold may be preferred for delirium prevention interventions that are resource intensive; this would correspond to a high negative predictive value and require fewer interventions to be performed. However, a higher specificity threshold comes at a cost in sensitivity: 83 of 191 cases of incident delirium (43.5%) were missed by the model with 90% specificity. Conversely, high sensitivity may be preferred for low-cost, low-risk interventions in which the goal is to capture all potential delirium cases, while acknowledging a higher NNS and the intervention being administered unnecessarily to more patients.

Our GBM model recovers many known delirium risk factors including advanced age, illness severity, functional or mobility impairment, alcohol misuse, and psychoactive or sedative drugs, and results were largely consistent between top-performing models.^1^ We excluded patients with delirium on presentation and obvious baseline cognitive dysfunction (ie, not oriented to person, time, or place) because these patients would receive delirium prevention measures without the need for a risk-assessment tool; therefore, a clear marker of dementia was not expected to be recovered in our model. Nevertheless, it is likely that some of the recovered variables are surrogates for baseline cognitive dysfunction, such as dependence for activities of daily living. The large sample size also allowed identification of variables less commonly associated with delirium, including nursing data fields (eg, urinary incontinence), vital signs, medications (eg, antimanic agents including lithium and valproic acid), and select comorbidities (eg, peripheral vascular disease).

Although delirium is usually considered to disproportionately affect the elderly, it also occurs in younger patients, with a prevalence of 4.7%^22^ and an incidence as high as 14% in high-risk groups.^23^ Unlike previous studies that focus only on older populations, our study does not exclude patients based on age. At the 90% specificity threshold, our GBM model predicted delirium correctly in patients as young as 22 years, with 46 of 114 of true positives (40.4%) in patients younger than 65 years, suggesting that our model is accurately predicting delirium, even in populations younger than those traditionally studied.

### Limitations

The incidence of delirium reported in our data set (4.8%) is lower than the national incidence (11%-14%). This discrepancy is likely due to the younger age (mean [SD] age, 57.1 [17.2] years) of our study population as well as the strict exclusion criteria of the study. Indeed, the rate of incident delirium in the overall cohort prior to application of exclusion criteria was 13.5%. The goal of this study was to develop a model to predict incident delirium within the hospital to implement preventive measures prior to delirium onset. Thus, our exclusion criteria were specifically chosen to eliminate any patients who were delirious on admission or known to have high risk of developing incident delirium. In practice, nonpharmacologic delirium prevention measures are already applied to both these subsets of patients. The high prevalence of delirium among excluded patients, which translates to an NNS of 2.7, suggests the exclusion criteria correctly identified the group of patients known to have an elevated risk.

It is possible that some cases of delirium were missed using the Nu-DESC because it was not performed, performed incorrectly, or performed correctly but with false-negative results. In addition, some cases were missed because several general medical units that have the highest rates of delirium only began routine delirium screening in January 2017.

Although they represent important risk factors for delirium, microbiology, radiology, pathology, and procedures were not included as potential predictors because of their high dimensionality or unavailability within the first 24 hours of admission. However, some of these risk factors may be inferred from other variables in our data set: for example, fever, leukocytosis, and treatment with anti-infective agents would suggest infection otherwise captured on blood cultures. Deliriogenic interventions such as feeding tubes, Foley catheters, and physical restraints are captured by our data set.

We recognize that newer predictive models such as ANNs have been shown to outperform older models such as GBM, RF, and LR in prediction accuracy.^24,25^ However, such models require more computational power and larger training data sets and are far more technically challenging to integrate into clinical workflow. With the goal of creating a usable clinical tool in mind, the use of simpler models is more appropriate for many institutions at this time. However, the use of more advanced models for delirium prediction remains promising and should be explored in the future. Ensemble learning techniques have been shown to boost performance in models trained using fewer predictors,^26,27^ but were not pursued because of computational constraints.

Incomplete EHR data, another limitation, was mitigated by explicitly modeling missing data through indicator variables, a method that was chosen for its simplicity and computational efficiency and has been shown to be effective for recurrent neural networks.^28^ Like recurrent neural networks, GBM, ANN, and RF can model interactions between missingness indicators and other observation inputs. However, linear models can only learn hard substitution rules with indicator variables and may provide biased results and lead to overfitting^29^; future experiments using alternative missing data methods such as imputation^30^ may yield better performance.

Our test set includes only hospital stays discharged between August 1, 2017, and November 30, 2017, and is derived from the same institution as our training set. Higher incidence of delirium has been reported during the winter, which may limit generalizability to other times of year.^31^ Notably, the incidence of delirium in our training and test sets is identical across the calendar year, and there is no evidence of seasonality of delirium in our cohort (eFigure 2 in the Supplement). Finally, we recognize that an external validation would provide valuable insight into how our model performs in other health systems. However, variation in delirium screening, data availability, and EHR capabilities limits the ability to immediately generalize our model to other health systems. Collecting a larger data set across multiple sites may help overcome overfitting and improve generalization of our model in the future.

## Conclusions

Our study demonstrates the feasibility of accurate incident delirium risk prediction from routine hospitalization data available in the EHR within 24 hours of admission and provides a list of putative delirium-related variables other institutions can use to develop their own models. Such a model may allow more precise targeting of delirium prevention resources to patients likely to benefit most.

## Notes

Supplement. **eFigure 1.** Prevalence of Comorbidities by Elixhauser Comorbidities Index in Train and Test Sets **eFigure 2.** Number of Included Hospital Stays (CSNs) by Month of Discharge **eFigure 3.** Area Under the Receiver Operating Characteristic Curve (AUC) for Machine Learning Models and AWOL Stratified by Age **eFigure 4.** Receiver Operating Characteristic (ROC) Curves for Machine Learning Models and AWOL Stratified by Age **eFigure 5.** Model Performance Using More Sensitive Delirium Outcome (Nu-DESC≥1) **eTable 1.** Continuous Predictor Characteristics **eTable 2.** Categorical Predictor Characteristics **eTable 3.** Confusion Matrix Metrics **eTable 4.** Confusion Matrix for Gradient Boosting Machine Using 90% Specificity Threshold **eTable 5.** Confusion Matrix for Gradient Boosting Machine Using 90% Sensitivity Threshold **eTable 6.** Confusion Matrix for Penalized Logistic Regression Using 90% Specificity Threshold **eTable 7.** Confusion Matrix for Penalized Logistic Regression Using 90% Sensitivity Threshold **eTable 8.** Confusion Matrix for Random Forest Using 90% Specificity Threshold **eTable 9.** Confusion Matrix for Random Forest Using 90% Sensitivity Threshold **eTable 10.** Confusion Matrix for AWOL Using AWOL≥2 Threshold
