# Supplementary material for: Development and Validation of an Electronic Health Record–Based Machine Learning Model to Estimate Delirium Risk in Newly Hospitalized Patients Without Known Cognitive Impairment
Source: JAMA Netw Open. 2018 Aug 3;1(4):e181018. doi: 10.1001/jamanetworkopen.2018.1018 (PMC6324291; doi:10.1001/jamanetworkopen.2018.1018)
Supplement: Supplement. — eFigure 1. Prevalence of Comorbidities by Elixhauser Comorbidities Index in Train and Test Sets eFigure 2. Number of Included Hospital Stays (CSNs) by Month of Discharge eFigure 3. Area Under the Receiver Operating Characteristic Curve (AUC) for Machine Learning Models and AWOL Stratified by Age eFigure 4. Receiver Operating Characteristic (ROC) Curves for Machine Learning Models and AWOL Stratified by Age eFigure 5. Model Performance Using More Sensitive Delirium Outcome (Nu-DESC≥1) eTable 1. Continuous Predictor Characteristics eTable 2. Categorical Predictor Characteristics eTable 3. Confusion Matrix Metrics eTable 4. Confusion Matrix for Gradient Boosting Machine Using 90% Specificity Threshold eTable 5. Confusion Matrix for Gradient Boosting Machine Using 90% Sensitivity Threshold eTable 6. Confusion Matrix for Penalized Logistic Regression Using 90% Specificity Threshold eTable 7. Confusion Matrix for Penalized Logistic Regression Using 90% Sensitivity Threshold eTable 8. Confusion Matrix for Random Forest Using 90% Specificity Threshold eTable 9. Confusion Matrix for Random Forest Using 90% Sensitivity Threshold eTable 10. Confusion Matrix for AWOL Using AWOL≥2 Threshold [file jamanetwopen-1-e181018-s001.pdf]

Supplementary Online Content

Wong A, Young AT, Liang AS, Gonzales R, Douglas VC, Hadley D. Development and Validation of an Electronic Health Record–Based Machine Learning Model to Estimate Delirium Risk in Newly Hospitalized Patients Without Known Cognitive Impairment. *JAMA Netw Open.* 2018;1(4):e181018. doi:10.1001/jamanetworkopen.2018.1018

eFigure 1. Prevalence of Comorbidities by Elixhauser Comorbidities Index in Train and Test Sets

eFigure 2. Number of Included Hospital Stays (CSNs) by Month of Discharge

eFigure 3. Area Under the Receiver Operating Characteristic Curve (AUC) for Machine Learning Models and AWOL Stratified by Age

eFigure 4. Receiver Operating Characteristic (ROC) Curves for Machine Learning Models and AWOL Stratified by Age

eFigure 5. Model Performance Using More Sensitive Delirium Outcome (Nu-DESC≥1)

eTable 1. Continuous Predictor Characteristics

eTable 2. Categorical Predictor Characteristics

eTable 3. Confusion Matrix Metrics

eTable 4. Confusion Matrix for Gradient Boosting Machine Using 90% Specificity Threshold

eTable 5. Confusion Matrix for Gradient Boosting Machine Using 90% Sensitivity Threshold

eTable 6. Confusion Matrix for Penalized Logistic Regression Using 90% Specificity Threshold

eTable 7. Confusion Matrix for Penalized Logistic Regression Using 90% Sensitivity Threshold

eTable 8. Confusion Matrix for Random Forest Using 90% Specificity Threshold

eTable 9. Confusion Matrix for Random Forest Using 90% Sensitivity Threshold

eTable 10. Confusion Matrix for AWOL Using AWOL≥2 Threshold

This supplementary material has been provided by the authors to give readers additional information about their work.

© 2018 Wong A et al. *JAMA Network Open.*

eFigure 1. Prevalence of comorbidities by Elixhauser Comorbidities Index in train and test sets. Tumor, solid tumor without metastasis; HTN, hypertension; FluidsLytes, fluid and electrolyte disorders; NeuroOther, other neurological disorders; CHF, congestive heart failure; DM, diabetes, uncomplicated; Mets, metastatic cancer; PVD, peripheral vascular disorders; PHTN, pulmonary circulation disorders; DMcx, diabetes, complicated; anemia, Deficiency anemia; PUD, peptic ulcer disease excluding bleeding; BloodLoss, blood loss anemia.

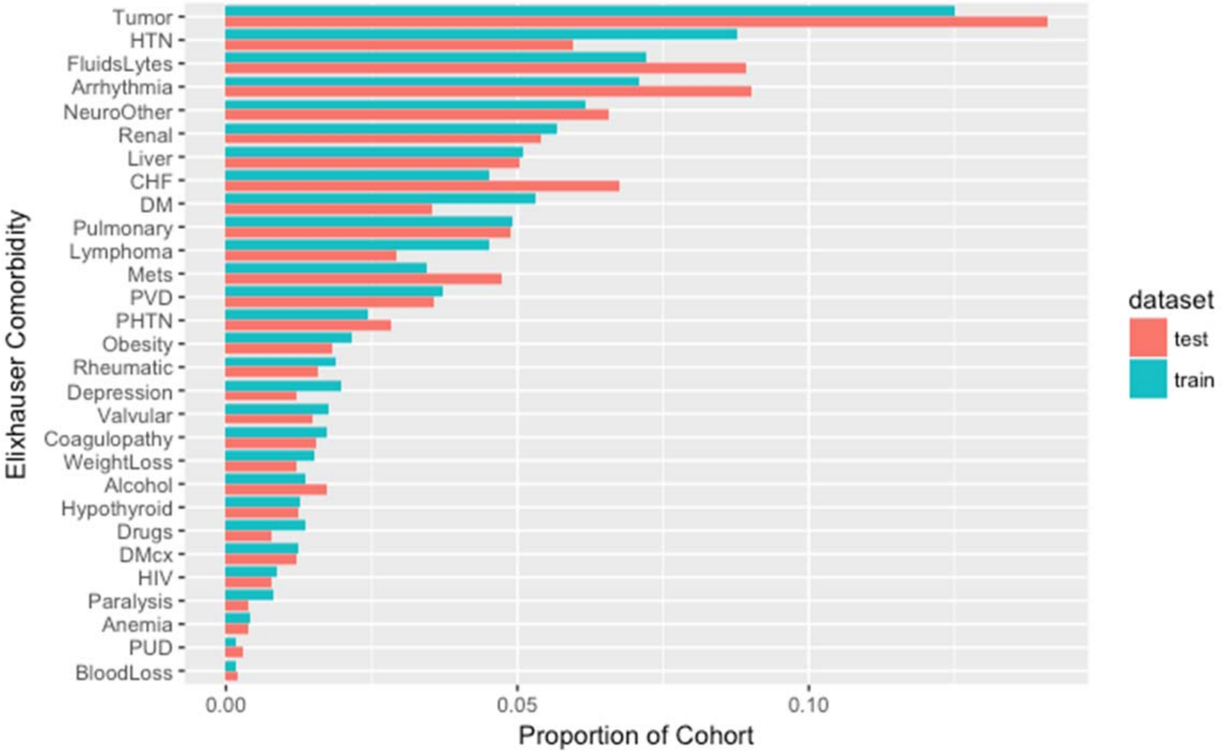

© 2018 Wong A et al. *JAMA Network Open.*

eFigure 2. Number of included hospital stays (CSNs) by month of discharge. Red and blue bars denote CSNs with an outcome of no delirium and delirium, respectively. A higher number of CSNs is recorded in 2017 in comparison with 2016 due to a wider implementation of screening. CSN, Contact Serial Number.

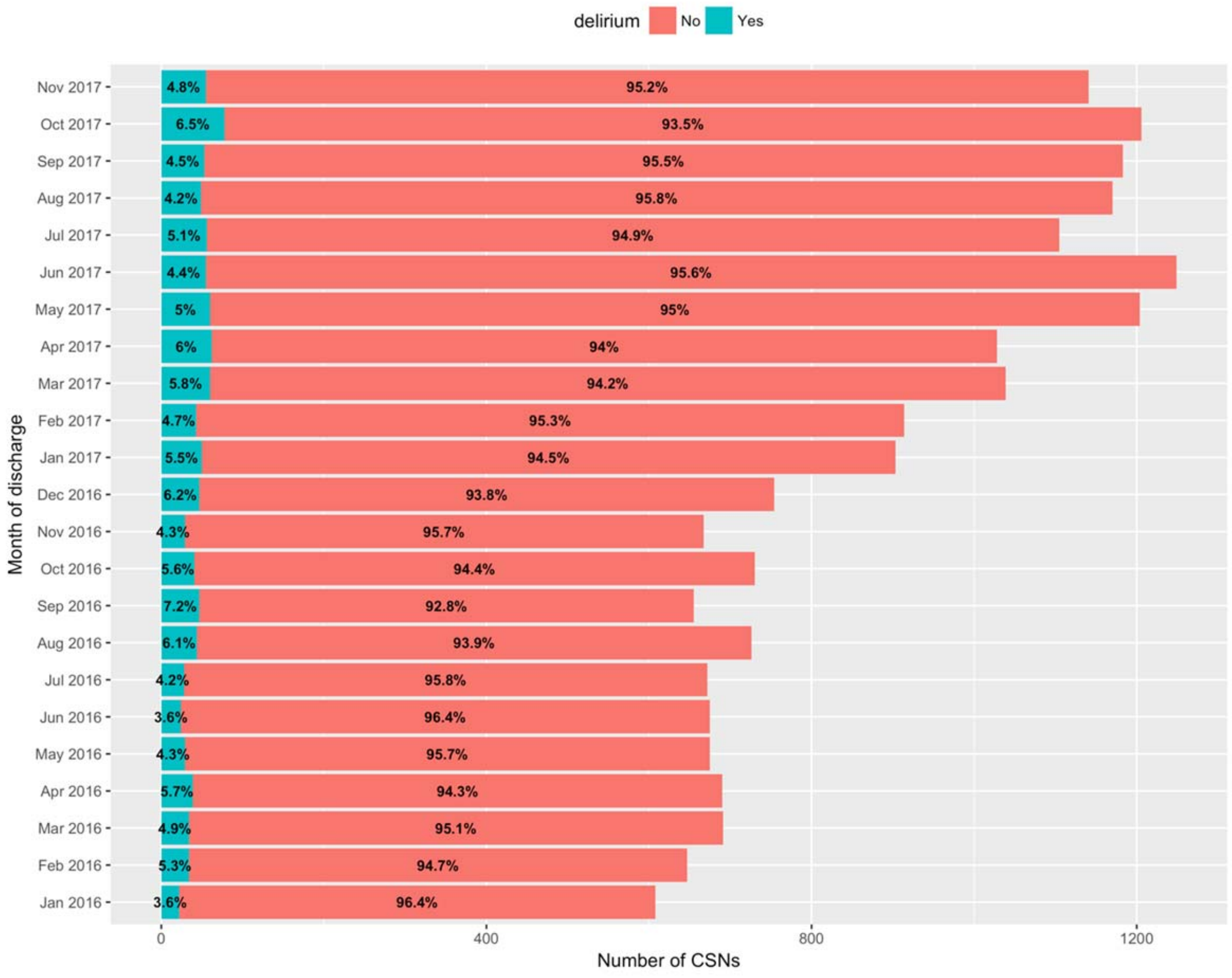

© 2018 Wong A et al. *JAMA Network Open.*

eFigure 3. Area under the receiver operating characteristic curve (AUC) for Machine Learning Models and AWOL stratified by age. Model performance was evaluated on a prospective test set (ROC curves shown are determined using the subset of the test set with AWOL measurements). AWOL=age, ability to spell world backwards, orientation, illness severity, GBM=gradient boosting machine, LR=penalized logistic regression, ANN=artificial neural network, RF=random forest, SVM=support vector machine.

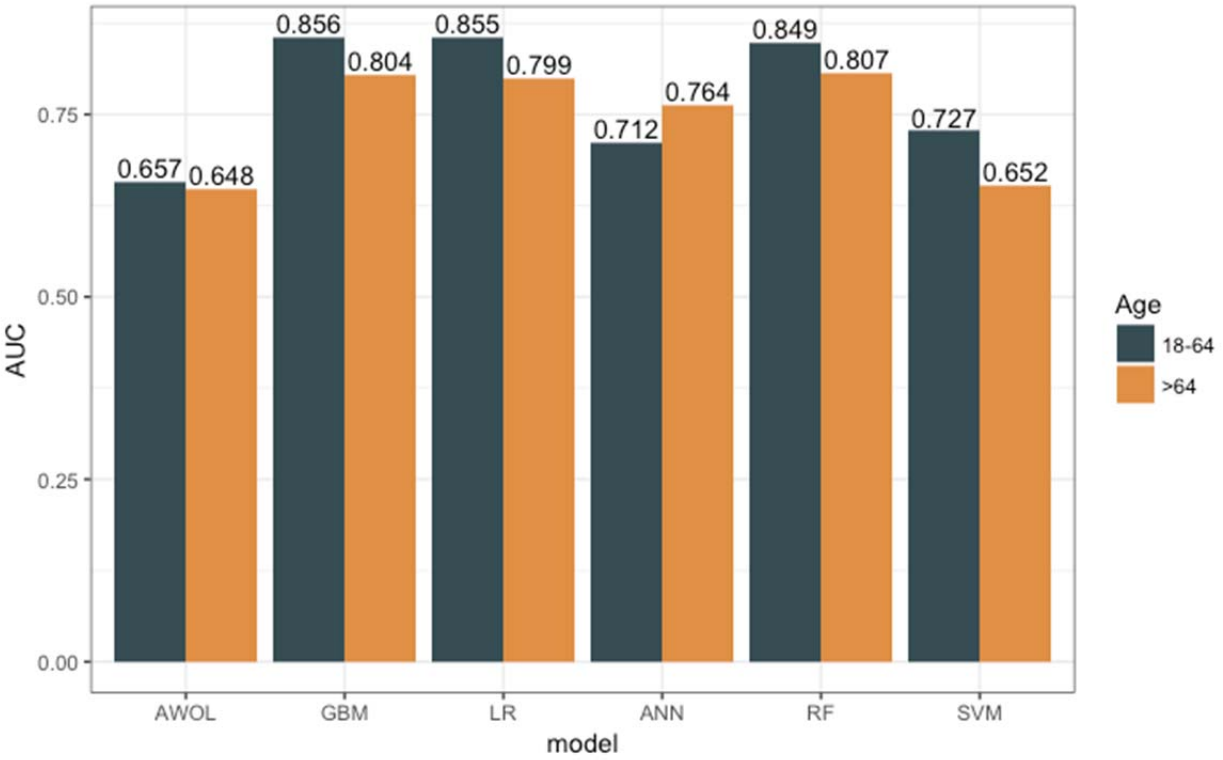

© 2018 Wong A et al. *JAMA Network Open.*

eFigure 4. Receiver Operating Characteristic (ROC) Curves for Machine Learning Models and AWOL stratified by age. Model performance was evaluated on a prospective test set (ROC curves shown are determined using the subset of the test set with AWOL measurements). AWOL=age, ability to spell world backwards, orientation, illness severity, GBM=gradient boosting machine, LR=penalized logistic regression, ANN=artificial neural network, RF=random forest, SVM=support vector machine.

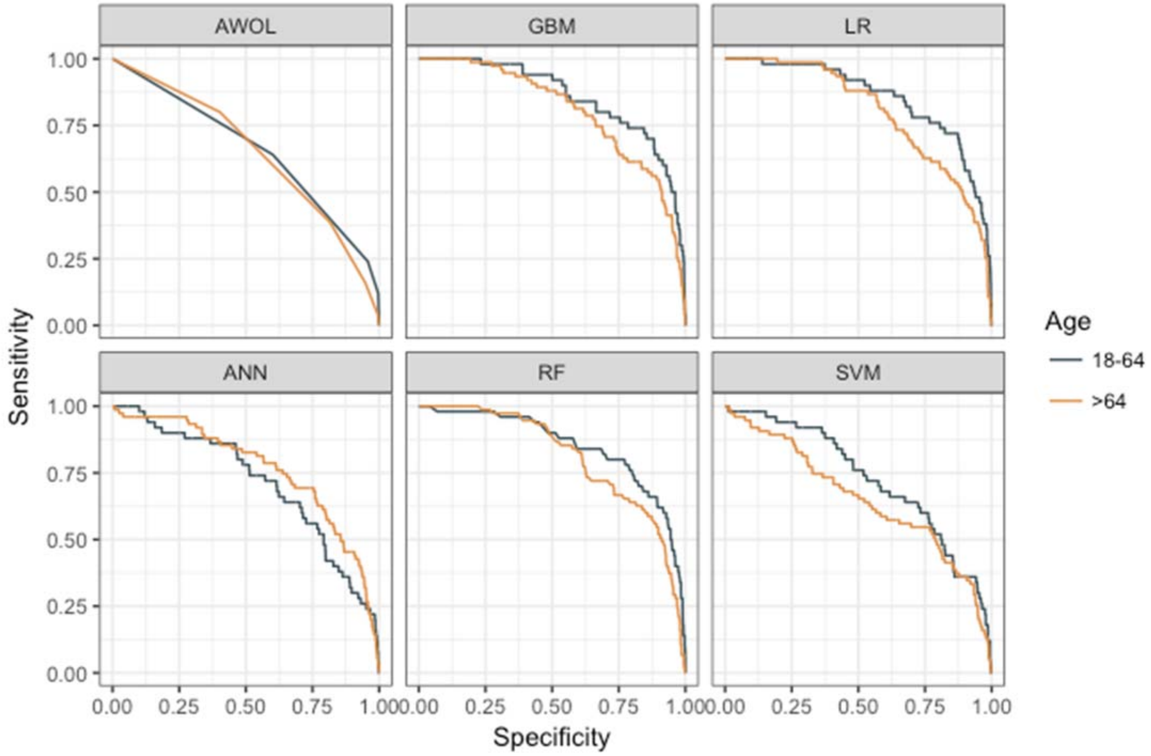

© 2018 Wong A et al. *JAMA Network Open.*

eFigure 5. Model performance using more sensitive delirium outcome (Nu-DESC≥1). The area under the receiver operating characteristic curve (AUC) is reported for each model on the complete test set as well as the subset of the test set with a valid AWOL score. AWOL, validated delirium risk screening tool based on Age, ability to spell WORLD backwards, Orientation, and iLlness severity; Nu-DESC, Nursing Delirium Screening Scale; GBM, gradient boosting machine; LR, penalized logistic regression; ANN, artificial neural network; RF, random forest; SVM, support vector machine.

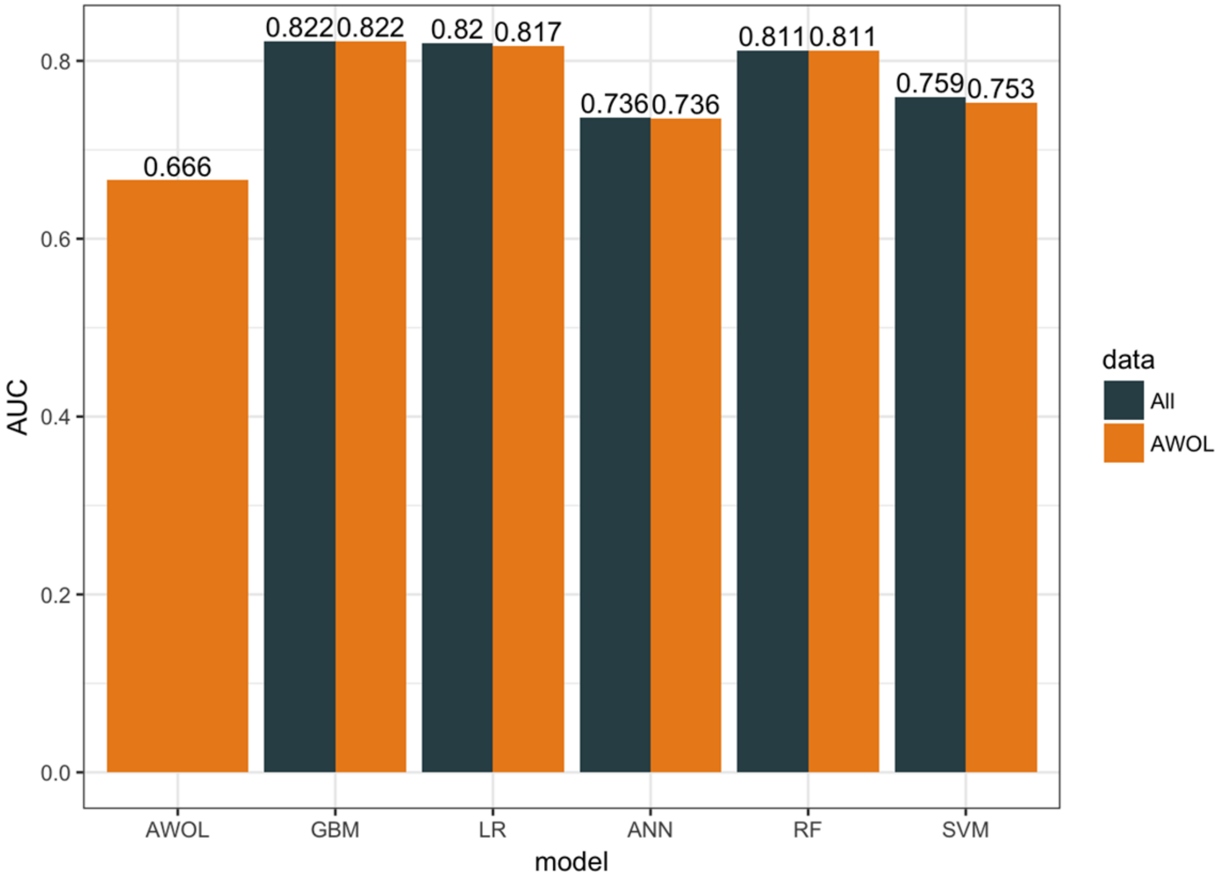

© 2018 Wong A et al. *JAMA Network Open.*

eTable 1. Continuous Predictor Characteristics in the train and test data sets. IQR, interquartile range; BMI, body mass index; ALT, alanine aminotransferase; AST, aspartate aminotransferase; days_since_onset, days since onset of pain; DBP, diastolic blood pressure; SBP, systolic blood pressure; Hgb, hemoglobin; Na, sodium; WBC, white blood cell; NR, nursing record; RR, respiratory rate.

	Variable	Train: Median (IQR)	Train: % missing	Test: Median (IQR)	Test: % missing
1	Admit Lab (AdLab) albumin	3.3 (2.7-3.7)	77.80%	3.3 (2.8-3.7)	75.5%
2	Admit: BMI	26.6 (22.8-31.5)	43.60%	26.5 (22.9-31.1)	49.7%
3	Admit: Height	66.0 (63.0-70.0)	38.50%	67.0 (63.0-70.0)	43.9%
4	Admit: Weight	2670.0 (2208.0-3213.0)	7.10%	2660.0 (2205.0-3185.0)	5.9%
5	Age	59.0 (46.0-69.0)	0.00%	60.0 (46.0-69.0)	0.0%
6	alkaline phosphatase	79.0 (61.0-115.0)	60.30%	77.0 (59.0-109.0)	56.5%
7	ALT	23.0 (16.0-40.0)	60.10%	22.0 (16.0-38.0)	56.2%
8	ammonia	35.0 (24.0-57.8)	99.10%	35.5 (25.2-48.2)	99.0%
9	arterial pH	7.4 (7.3-7.4)	66.10%	7.4 (7.3-7.4)	62.3%
10	AST	27.0 (20.0-45.0)	60.10%	27.0 (20.0-44.0)	56.2%
11	bicarbonate	25.0 (23.0-27.0)	66.10%	25.0 (23.0-27.0)	62.3%
12	calcium	8.7 (8.3-9.1)	20.50%	8.8 (8.4-9.2)	15.6%
13	chloride	103.0 (100.0-106.0)	19.40%	103.0 (100.0-106.0)	14.9%
14	days_since_onset	2.0 (0.0-43.5)	69.20%	2.0 (0.0-28.0)	70.7%
15	First DBP	60.0 (54.0-66.0)	0.00%	60.0 (54.0-67.0)	0.0%
16	First SBP	102.0 (100.0-108.0)	0.00%	102.0 (100.0-108.0)	0.0%
17	hematocrit	35.5 (31.1-39.5)	16.40%	35.5 (31.0-39.7)	12.6%
18	Max arterial pO2	47.0 (34.0-81.0)	67.40%	46.0 (34.0-70.0)	64.0%
19	Max BUN	14.0 (10.0-21.0)	21.00%	14.0 (10.0-21.0)	16.6%
20	Max creatinine	0.9 (0.7-1.1)	18.60%	0.8 (0.7-1.1)	14.1%
21	Max glucose	121.0 (103.0-151.0)	67.40%	121.0 (103.0-150.0)	15.2%
22	Max Hgb	11.7 (10.1-13.2)	17.70%	11.8 (10.2-13.4)	13.2%
23	Max lactate	1.6 (1.1-2.7)	62.80%	1.6 (1.1-2.7)	60.0%
24	Max Na	138.0 (136.0-140.0)	18.80%	137.0 (135.0-139.0)	14.5%
25	Max WBC	9.2 (6.8-12.4)	17.70%	9.1 (6.8-12.1)	13.2%

© 2018 Wong A et al. *JAMA Network Open.*

26	Min arterial pO2	45.0 (30.0-155.0)	91.00%	37.0 (28.0-63.5)	89.8%
27	Min BUN	13.0 (9.0-22.0)	63.50%	14.0 (10.0-23.0)	59.0%
28	Min creatinine	0.8 (0.7-1.1)	58.30%	0.8 (0.6-1.2)	53.0%
29	Min glucose	101.0 (90.0-121.0)	85.00%	101.0 (89.0-121.0)	53.3%
30	Min Hgb	10.6 (8.9-12.1)	57.30%	10.8 (9.0-12.3)	52.6%
31	Min lactate	1.3 (0.9-1.9)	87.50%	1.3 (1.0-1.7)	86.0%
32	Min Na	136.0 (133.0-138.0)	63.80%	135.0 (132.0-137.0)	58.8%
33	Min WBC	7.6 (5.4-10.4)	56.90%	7.4 (5.5-10.1)	52.1%
34	NR avg DBP	60.0 (54.0-67.0)	6.20%	61.0 (55.0-67.0)	5.5%
35	NR avg SBP	102.0 (100.0-109.0)	6.20%	103.0 (100.0-109.0)	5.5%
36	platelet	219.0 (168.0-282.0)	17.70%	222.0 (169.0-284.0)	13.2%
37	Pulse	72.0 (59.0-100.0)	6.20%	71.0 (59.0-100.0)	5.5%
38	RR	13.0 (11.0-16.0)	6.20%	14.0 (11.0-16.0)	5.5%
39	serum potassium	3.9 (3.6-4.3)	19.10%	3.9 (3.6-4.2)	14.7%
40	SpO2	100.0 (100.0-100.0)	6.20%	100.0 (100.0-100.0)	5.5%
41	Temp	97.2 (96.8-98.1)	6.10%	97.3 (96.8-97.9)	5.5%
42	Total bilirubin	0.8 (0.5-1.2)	60.10%	0.8 (0.6-1.2)	55.5%

© 2018 Wong A et al. *JAMA Network Open.*

eTable 2. Categorical Predictor characteristics in the train and test data sets. NA, non-applicable; NG tube, nasogastric tube; AdMed, admission medications; HomeMed, home medications.

	Variable	Level	Train: No. (%)	Test: No. (%)
1	Gender	Female	7335 (51.6%)	1966 (49.2%)
2	Gender	Male	6892 (48.4%)	2030 (50.8%)
3	Gender	Unknown/Declined	0 (0.0%)	0 (0.0%)
4	Race	White or Caucasian	8372 (58.8%)	2320 (58.1%)
5	Race	Other	2160 (15.2%)	640 (16.0%)
6	Race	Asian	1751 (12.3%)	514 (12.9%)
7	Race	Black or African American	1443 (10.1%)	391 (9.8%)
8	Race	Unknown/Declined	133 (0.9%)	47 (1.2%)
9	Race	Native Hawaiian or Other Pacific Islander	127 (0.9%)	34 (0.9%)
10	Race	(Other)	241 (1.7%)	50 (1.3%)
11	Ethnicity	Hispanic or Latino	1818 (12.8%)	536 (13.4%)
12	Ethnicity	Not Hispanic or Latino	12113 (85.1%)	3391 (84.9%)
13	Ethnicity	Unknown/Declined	296 (2.1%)	69 (1.7%)
14	Ethnicity	NA	0 (0.0%)	0 (0.0%)
15	Marital Status	Married	6620 (46.5%)	1899 (47.5%)
16	Marital Status	Separated	1255 (8.8%)	327 (8.2%)
17	Marital Status	Significant Other	266 (1.9%)	77 (1.9%)
18	Marital Status	Single	4891 (34.4%)	1370 (34.3%)
19	Marital Status	Unknown/Declined	201 (1.4%)	68 (1.7%)
20	Marital Status	Widowed	994 (7.0%)	255 (6.4%)
21	Marital Status	NA	0 (0.0%)	0 (0.0%)
22	Payor	CALIF DEPT CORRECTIONS	42 (0.3%)	5 (0.1%)
23	Payor	CVD CA	855 (6.0%)	239 (6.0%)
24	Payor	HMO/SENIOR	443 (3.1%)	117 (2.9%)
25	Payor	MEDI-CAL	3063 (21.5%)	829 (20.7%)
26	Payor	MEDICARE	5686 (40.0%)	1621 (40.6%)
27	Payor	OTHER	4070 (28.6%)	1167 (29.2%)
28	Payor	TRANSPLANT	68 (0.5%)	18 (0.5%)
29	*Dressing	Dependent	184 (1.3%)	62 (1.6%)
30	*Dressing	Independent	9140 (64.2%)	2710 (67.8%)
31	*Dressing	Needs assistance	3634 (25.5%)	981 (24.5%)
32	*Dressing	Unable to assess	17 (0.1%)	1 (0.0%)
33	*Dressing	NA	1252 (8.8%)	242 (6.1%)
34	*Grooming	Dependent	173 (1.2%)	57 (1.4%)
35	*Grooming	Independent	9942 (69.9%)	2898 (72.5%)
36	*Grooming	Needs assistance	2839 (20.0%)	798 (20.0%)

© 2018 Wong A et al. *JAMA Network Open.*

37	*Grooming	Unable to assess	16 (0.1%)	1 (0.0%)
38	*Grooming	NA	1257 (8.8%)	242 (6.1%)
39	*Feeding	Dependent	114 (0.8%)	40 (1.0%)
40	*Feeding	Independent	11630 (81.7%)	3378 (84.5%)
41	*Feeding	Needs assistance	1211 (8.5%)	334 (8.4%)
42	*Feeding	Unable to assess	24 (0.2%)	2 (0.1%)
43	*Feeding	NA	1248 (8.8%)	242 (6.1%)
44	*Bathing	Dependent	206 (1.4%)	71 (1.8%)
45	*Bathing	Independent	8814 (62.0%)	2592 (64.9%)
46	*Bathing	Needs assistance	3920 (27.6%)	1090 (27.3%)
47	*Bathing	Unable to assess	20 (0.1%)	1 (0.0%)
48	*Bathing	NA	1267 (8.9%)	242 (6.1%)
49	*Toileting	Dependent	204 (1.4%)	63 (1.6%)
50	*Toileting	Independent	8701 (61.2%)	2375 (59.4%)
51	*Toileting	Needs assistance	4034 (28.4%)	1017 (25.5%)
52	*Toileting	Unable to assess	16 (0.1%)	1 (0.0%)
53	*Toileting	NA	1272 (8.9%)	540 (13.5%)
54	*In/Out Bed	Dependent	250 (1.8%)	75 (1.9%)
55	*In/Out Bed	Independent	8414 (59.1%)	2304 (57.7%)
56	*In/Out Bed	Needs assistance	4239 (29.8%)	1071 (26.8%)
57	*In/Out Bed	Unable to assess	33 (0.2%)	6 (0.2%)
58	*In/Out Bed	NA	1291 (9.1%)	540 (13.5%)
59	*Weakness of Arms/Hands	Both	1144 (8.0%)	370 (9.3%)
60	*Weakness of Arms/Hands	Left	450 (3.2%)	148 (3.7%)
61	*Weakness of Arms/Hands	None	10820 (76.1%)	3112 (77.9%)
62	*Weakness of Arms/Hands	Right	407 (2.9%)	122 (3.1%)
63	*Weakness of Arms/Hands	Unable to assess	26 (0.2%)	3 (0.1%)
64	*Weakness of Arms/Hands	NA	1380 (9.7%)	241 (6.0%)
65	*Weakness of Legs	Both	2886 (20.3%)	919 (23.0%)
66	*Weakness of Legs	Left	1051 (7.4%)	283 (7.1%)
67	*Weakness of Legs	None	8055 (56.6%)	2334 (58.4%)
68	*Weakness of Legs	Right	864 (6.1%)	210 (5.3%)
69	*Weakness of Legs	Unable to assess	55 (0.4%)	9 (0.2%)
70	*Weakness of Legs	NA	1316 (9.3%)	241 (6.0%)
71	*Bowel or Bladder Habits	No	11274 (79.2%)	3432 (85.9%)
72	*Bowel or Bladder Habits	Unable to assess	104 (0.7%)	26 (0.7%)
73	*Bowel or Bladder Habits	Yes	1080 (7.6%)	295 (7.4%)
74	*Bowel or Bladder Habits	NA	1769 (12.4%)	243 (6.1%)
75	*Hearing Right Ear	Functional	11454 (80.5%)	1927 (48.2%)

© 2018 Wong A et al. *JAMA Network Open.*

76	*Hearing Right Ear	NA	1594 (11.2%)	1440 (36.0%)
77	*Hearing Right Ear	Poor hearing	1048 (7.4%)	243 (6.1%)
78	*Hearing Right Ear	Difficulty with noise	59 (0.4%)	205 (5.1%)
79	*Hearing Right Ear	Deaf	51 (0.4%)	162 (4.1%)
80	*Hearing Right Ear	Unable to assess	21 (0.1%)	12 (0.3%)
81	*Hearing Right Ear	(Other)	0 (0.0%)	7 (0.2%)
82	*Hearing Left Ear	Functional	11478 (80.7%)	1920 (48.0%)
83	*Hearing Left Ear	NA	1545 (10.9%)	1440 (36.0%)
84	*Hearing Left Ear	Poor hearing	1073 (7.5%)	243 (6.1%)
85	*Hearing Left Ear	Difficulty with noise	60 (0.4%)	216 (5.4%)
86	*Hearing Left Ear	Deaf	51 (0.4%)	157 (3.9%)
87	*Hearing Left Ear	Unable to assess	20 (0.1%)	15 (0.4%)
88	*Hearing Left Ear	(Other)	0 (0.0%)	5 (0.1%)
89	*Vision	Functional	7758 (54.5%)	2107 (52.7%)
90	*Vision	Poor Eyesight	4710 (33.1%)	1341 (33.6%)
91	*Vision	Unable to Assess	26 (0.2%)	5 (0.1%)
92	*Vision	NA	1733 (12.2%)	543 (13.6%)
93	Foley	[REMOVED] INDWELLING URINARY CATHETER	5826 (41.0%)	1424 (35.6%)
94	Foley	INDWELLING URINARY CATHETER	320 (2.2%)	86 (2.2%)
95	Foley	NA	8081 (56.8%)	2486 (62.2%)
96	IV 1	Peripheral IV	10144 (71.3%)	3047 (76.3%)
97	IV 1	NA	4083 (28.7%)	949 (23.7%)
98	NG tube	[REMOVED] FEEDING TUBE	54 (0.4%)	10 (0.3%)
99	NG tube	FEEDING TUBE	7 (0.0%)	1 (0.0%)
100	NG tube	NA	14166 (99.6%)	3985 (99.7%)
101	Chest tube	[REMOVED] CHEST TUBE/DRAIN CHEST	191 (1.3%)	86 (2.2%)
102	Chest tube	NA	14036 (98.7%)	3910 (97.8%)
103	*Mobility	0 - Ambulates w/no gait disturbance	10226 (71.9%)	2653 (66.4%)
104	*Mobility	0 - Unable to ambulate or transfer	1341 (9.4%)	335 (8.4%)
105	*Mobility	1 - Ambulates or transfers w/assist device	1758 (12.4%)	481 (12.0%)
106	*Mobility	1 - Ambulates w/unsteady gait, no assist device	32 (0.2%)	14 (0.4%)
107	*Mobility	NA	870 (6.1%)	513 (12.8%)
108	*Elimination	0 - Independent	10804 (75.9%)	2786 (69.7%)
109	*Elimination	1 - Incontinence	602 (4.2%)	152 (3.8%)
110	*Elimination	1 - Independent with frequency or diarrhea	87 (0.6%)	23 (0.6%)

© 2018 Wong A et al. *JAMA Network Open.*

111	*Elimination	1 - Need assist with toilet	1864 (13.1%)	522 (13.1%)
112	*Elimination	NA	870 (6.1%)	513 (12.8%)
113	*History of Falls	0 - No	12418 (87.3%)	3211 (80.4%)
114	*History of Falls	1 - Unknown	391 (2.7%)	111 (2.8%)
115	*History of Falls	1 - Yes, before admission	548 (3.9%)	161 (4.0%)
116	*History of Falls	NA	870 (6.1%)	513 (12.8%)
117	*Current Medications	0 - None of the above	12401 (87.2%)	3184 (79.7%)
118	*Current Medications	1 - Anticonvulsants, Tranquilizers, Hypnotics, Psychotropics	956 (6.7%)	299 (7.5%)
119	*Current Medications	NA	870 (6.1%)	513 (12.8%)
120	*Schmid Fall Score	0	7472 (52.5%)	1915 (47.9%)
121	*Schmid Fall Score	1	3238 (22.8%)	834 (20.9%)
122	*Schmid Fall Score	2	2024 (14.2%)	545 (13.6%)
123	*Schmid Fall Score	3	564 (4.0%)	170 (4.3%)
124	*Schmid Fall Score	4	59 (0.4%)	19 (0.5%)
125	*Schmid Fall Score	5	0 (0.0%)	0 (0.0%)
126	*Schmid Fall Score	NA	870 (6.1%)	513 (12.8%)
127	*Do you have pain or discomfort?	No Pain	4418 (31.1%)	1414 (35.4%)
128	*Do you have pain or discomfort?	Yes, new onset	3285 (23.1%)	902 (22.6%)
129	*Do you have pain or discomfort?	Yes, ongoing	5534 (38.9%)	1441 (36.1%)
130	*Do you have pain or discomfort?	NA	990 (7.0%)	239 (6.0%)
131	*Have you had any unplanned weight loss?	No	11493 (80.8%)	3273 (81.9%)
132	*Have you had any unplanned weight loss?	Yes (Comment)	1517 (10.7%)	482 (12.1%)
133	*Have you had any unplanned weight loss?	NA	1217 (8.6%)	241 (6.0%)
134	*Difficulty Chewing	No	12451 (87.5%)	3566 (89.2%)
135	*Difficulty Chewing	Unable to assess	34 (0.2%)	4 (0.1%)
136	*Difficulty Chewing	Yes	657 (4.6%)	184 (4.6%)
137	*Difficulty Chewing	NA	1085 (7.6%)	242 (6.1%)
138	*Difficulty Swallowing	No	12407 (87.2%)	3541 (88.6%)
139	*Difficulty Swallowing	Yes	753 (5.3%)	215 (5.4%)
140	*Difficulty Swallowing	NA	1067 (7.5%)	240 (6.0%)
141	*Tube Feeding	No	12998 (91.4%)	3725 (93.2%)
142	*Tube Feeding	Yes	143 (1.0%)	32 (0.8%)
143	*Tube Feeding	NA	1086 (7.6%)	239 (6.0%)
144	*Total Parenteral Nutrition (TPN)	No	13036 (91.6%)	3733 (93.4%)

© 2018 Wong A et al. *JAMA Network Open.*

145	*Total Parenteral Nutrition (TPN)	Yes	98 (0.7%)	24 (0.6%)
146	*Total Parenteral Nutrition (TPN)	NA	1093 (7.7%)	239 (6.0%)
147	*Pressure Ulcer	No	12830 (90.2%)	3667 (91.8%)
148	*Pressure Ulcer	Yes	275 (1.9%)	87 (2.2%)
149	*Pressure Ulcer	NA	1122 (7.9%)	242 (6.1%)
150	*Non Healing Wound	No	12479 (87.7%)	3577 (89.5%)
151	*Non Healing Wound	Yes	619 (4.4%)	179 (4.5%)
152	*Non Healing Wound	NA	1129 (7.9%)	240 (6.0%)
153	*Dietitian Consult Needed	No	11855 (83.3%)	3401 (85.1%)
154	*Dietitian Consult Needed	Yes	1173 (8.2%)	355 (8.9%)
155	*Dietitian Consult Needed	NA	1199 (8.4%)	240 (6.0%)
156	Admit Alcohol use	No	5511 (38.7%)	1470 (36.8%)
157	Admit Alcohol use	Not Asked	115 (0.8%)	33 (0.8%)
158	Admit Alcohol use	Yes	3788 (26.6%)	992 (24.8%)
159	Admit Alcohol use	NA	4813 (33.8%)	1501 (37.6%)
160	Admit Smoking Status	Never	5177 (36.4%)	1383 (34.6%)
161	Admit Smoking Status	Not Asked	125 (0.9%)	36 (0.9%)
162	Admit Smoking Status	Passive	52 (0.4%)	20 (0.5%)
163	Admit Smoking Status	Quit	3293 (23.1%)	855 (21.4%)
164	Admit Smoking Status	Yes	1125 (7.9%)	315 (7.9%)
165	Admit Smoking Status	NA	4455 (31.3%)	1387 (34.7%)
166	Admit drug use	No	7440 (52.3%)	1928 (48.2%)
167	Admit drug use	Not Asked	159 (1.1%)	21 (0.5%)
168	Admit drug use	Yes	1601 (11.3%)	469 (11.7%)
169	Admit drug use	NA	5027 (35.3%)	1578 (39.5%)
170	Best Eye Response	1	9 (0.1%)	4 (0.1%)
171	Best Eye Response	2	45 (0.3%)	5 (0.1%)
172	Best Eye Response	3	1108 (7.8%)	261 (6.5%)
173	Best Eye Response	4	6117 (43.0%)	1797 (45.0%)
174	Best Eye Response	NA	6948 (48.8%)	1929 (48.3%)
175	Best Verbal Response	1	0 (0.0%)	0 (0.0%)
176	Best Verbal Response	2	0 (0.0%)	0 (0.0%)
177	Best Verbal Response	3	0 (0.0%)	0 (0.0%)
178	Best Verbal Response	4	800 (5.6%)	203 (5.1%)
179	Best Verbal Response	5	6478 (45.5%)	1866 (46.7%)
180	Best Verbal Response	NA	6949 (48.8%)	1927 (48.2%)
181	Best Motor Response (upper extremities)	1	3 (0.0%)	4 (0.1%)

© 2018 Wong A et al. *JAMA Network Open.*

182	Best Motor Response (upper extremities)	2	1 (0.0%)	1 (0.0%)
183	Best Motor Response (upper extremities)	3	7 (0.0%)	0 (0.0%)
184	Best Motor Response (upper extremities)	4	17 (0.1%)	4 (0.1%)
185	Best Motor Response (upper extremities)	5	152 (1.1%)	28 (0.7%)
186	Best Motor Response (upper extremities)	6	7099 (49.9%)	2033 (50.9%)
187	Best Motor Response (upper extremities)	NA	6948 (48.8%)	1926 (48.2%)
188	AdMed_alpha-Adrenergic Blocking Agents	0	2050 (14.4%)	1305 (32.7%)
189	AdMed_alpha-Adrenergic Blocking Agents	1	9 (0.1%)	7 (0.2%)
190	AdMed_alpha-Adrenergic Blocking Agents	NA	12168 (85.5%)	2684 (67.2%)
191	AdMed_Analgesics and Antipyretics	0	1106 (7.8%)	644 (16.1%)
192	AdMed_Analgesics and Antipyretics	1	953 (6.7%)	668 (16.7%)
193	AdMed_Analgesics and Antipyretics	NA	12168 (85.5%)	2684 (67.2%)
194	AdMed_Anorexigenic Agents and Respiratory and Cerebral Stimulants	0	2059 (14.5%)	1311 (32.8%)
195	AdMed_Anorexigenic Agents and Respiratory and Cerebral Stimulants	1	0 (0.0%)	1 (0.0%)
196	AdMed_Anorexigenic Agents and Respiratory and Cerebral Stimulants	NA	12168 (85.5%)	2684 (67.2%)
197	AdMed_Anti-infective Agents	0	1874 (13.2%)	1157 (29.0%)
198	AdMed_Anti-infective Agents	1	185 (1.3%)	155 (3.9%)
199	AdMed_Anti-infective Agents	NA	12168 (85.5%)	2684 (67.2%)
200	AdMed_Anticholinergic Agents	0	2035 (14.3%)	1289 (32.3%)
201	AdMed_Anticholinergic Agents	1	24 (0.2%)	23 (0.6%)
202	AdMed_Anticholinergic Agents	NA	12168 (85.5%)	2684 (67.2%)
203	AdMed_Anticonvulsants	0	1727 (12.1%)	1191 (29.8%)
204	AdMed_Anticonvulsants	1	332 (2.3%)	121 (3.0%)
205	AdMed_Anticonvulsants	NA	12168 (85.5%)	2684 (67.2%)
206	AdMed_Antilipemic Agents	0	2047 (14.4%)	1292 (32.3%)
207	AdMed_Antilipemic Agents	1	12 (0.1%)	20 (0.5%)
208	AdMed_Antilipemic Agents	NA	12168 (85.5%)	2684 (67.2%)
209	AdMed_Antimanic Agents	0	2056 (14.5%)	1311 (32.8%)

© 2018 Wong A et al. *JAMA Network Open.*

210	AdMed_Antimanic Agents	1	3 (0.0%)	1 (0.0%)
211	AdMed_Antimanic Agents	NA	12168 (85.5%)	2684 (67.2%)
212	AdMed_Antimigraine Agents	0	2040 (14.3%)	1287 (32.2%)
213	AdMed_Antimigraine Agents	1	19 (0.1%)	25 (0.6%)
214	AdMed_Antimigraine Agents	NA	12168 (85.5%)	2684 (67.2%)
215	AdMed_Antineoplastic Agents	0	2022 (14.2%)	1297 (32.5%)
216	AdMed_Antineoplastic Agents	1	37 (0.3%)	15 (0.4%)
217	AdMed_Antineoplastic Agents	NA	12168 (85.5%)	2684 (67.2%)
218	AdMed_Antiparkinsonian Agents	0	2058 (14.5%)	1312 (32.8%)
219	AdMed_Antiparkinsonian Agents	1	1 (0.0%)	0 (0.0%)
220	AdMed_Antiparkinsonian Agents	NA	12168 (85.5%)	2684 (67.2%)
221	AdMed_Anxiolytics, Sedatives, and Hypnotics	0	1936 (13.6%)	1238 (31.0%)
222	AdMed_Anxiolytics, Sedatives, and Hypnotics	1	123 (0.9%)	74 (1.9%)
223	AdMed_Anxiolytics, Sedatives, and Hypnotics	NA	12168 (85.5%)	2684 (67.2%)
224	AdMed_Autonomic Drugs, Miscellaneous	0	2039 (14.3%)	1292 (32.3%)
225	AdMed_Autonomic Drugs, Miscellaneous	1	20 (0.1%)	20 (0.5%)
226	AdMed_Autonomic Drugs, Miscellaneous	NA	12168 (85.5%)	2684 (67.2%)
227	AdMed_beta-Adrenergic Blocking Agents	0	2039 (14.3%)	1294 (32.4%)
228	AdMed_beta-Adrenergic Blocking Agents	1	20 (0.1%)	18 (0.5%)
229	AdMed_beta-Adrenergic Blocking Agents	NA	12168 (85.5%)	2684 (67.2%)
230	AdMed_Blood Derivatives	0	2057 (14.5%)	1311 (32.8%)
231	AdMed_Blood Derivatives	1	2 (0.0%)	1 (0.0%)
232	AdMed_Blood Derivatives	NA	12168 (85.5%)	2684 (67.2%)
233	AdMed_Blood Formation, Coagulation, and Thrombosis	0	1630 (11.5%)	863 (21.6%)
234	AdMed_Blood Formation, Coagulation, and Thrombosis	1	429 (3.0%)	449 (11.2%)
235	AdMed_Blood Formation, Coagulation, and Thrombosis	NA	12168 (85.5%)	2684 (67.2%)
236	AdMed_Calcium-Channel Blocking Agents	0	2054 (14.4%)	1304 (32.6%)

© 2018 Wong A et al. *JAMA Network Open.*

237	AdMed_Calcium-Channel Blocking Agents	1	5 (0.0%)	8 (0.2%)
238	AdMed_Calcium-Channel Blocking Agents	NA	12168 (85.5%)	2684 (67.2%)
239	AdMed_Cardiac Drugs	0	2014 (14.2%)	1285 (32.2%)
240	AdMed_Cardiac Drugs	1	45 (0.3%)	27 (0.7%)
241	AdMed_Cardiac Drugs	NA	12168 (85.5%)	2684 (67.2%)
242	AdMed_Devices	0	861 (6.1%)	168 (4.2%)
243	AdMed_Devices	1	1198 (8.4%)	1144 (28.6%)
244	AdMed_Devices	NA	12168 (85.5%)	2684 (67.2%)
245	AdMed_Diagnostic Agents	0	2023 (14.2%)	1293 (32.4%)
246	AdMed_Diagnostic Agents	1	36 (0.3%)	19 (0.5%)
247	AdMed_Diagnostic Agents	NA	12168 (85.5%)	2684 (67.2%)
248	AdMed_Electrolytic, Caloric, and Water Balance	0	811 (5.7%)	154 (3.9%)
249	AdMed_Electrolytic, Caloric, and Water Balance	1	1248 (8.8%)	1158 (29.0%)
250	AdMed_Electrolytic, Caloric, and Water Balance	NA	12168 (85.5%)	2684 (67.2%)
251	AdMed_Enzymes	0	2053 (14.4%)	1312 (32.8%)
252	AdMed_Enzymes	1	6 (0.0%)	0 (0.0%)
253	AdMed_Enzymes	NA	12168 (85.5%)	2684 (67.2%)
254	AdMed_Eye, Ear, Nose, and Throat (EENT) Preparations	0	1939 (13.6%)	1247 (31.2%)
255	AdMed_Eye, Ear, Nose, and Throat (EENT) Preparations	1	120 (0.8%)	65 (1.6%)
256	AdMed_Eye, Ear, Nose, and Throat (EENT) Preparations	NA	12168 (85.5%)	2684 (67.2%)
257	AdMed_Fibromyalgia Agents	0	2058 (14.5%)	1309 (32.8%)
258	AdMed_Fibromyalgia Agents	1	1 (0.0%)	3 (0.1%)
259	AdMed_Fibromyalgia Agents	NA	12168 (85.5%)	2684 (67.2%)
260	AdMed_First Generation Antihistamines	0	1989 (14.0%)	1260 (31.5%)
261	AdMed_First Generation Antihistamines	1	70 (0.5%)	52 (1.3%)
262	AdMed_First Generation Antihistamines	NA	12168 (85.5%)	2684 (67.2%)
263	AdMed_Gastrointestinal Drugs	0	1379 (9.7%)	641 (16.0%)
264	AdMed_Gastrointestinal Drugs	1	680 (4.8%)	671 (16.8%)

© 2018 Wong A et al. *JAMA Network Open.*

265	AdMed_Gastrointestinal Drugs	NA	12168 (85.5%)	2684 (67.2%)
266	AdMed_General Anesthetics	0	2058 (14.5%)	1311 (32.8%)
267	AdMed_General Anesthetics	1	1 (0.0%)	1 (0.0%)
268	AdMed_General Anesthetics	NA	12168 (85.5%)	2684 (67.2%)
269	AdMed_Hormones and Synthetic Substitutes	0	1727 (12.1%)	1134 (28.4%)
270	AdMed_Hormones and Synthetic Substitutes	1	332 (2.3%)	178 (4.5%)
271	AdMed_Hormones and Synthetic Substitutes	NA	12168 (85.5%)	2684 (67.2%)
272	AdMed_Hypotensive Agents	0	2014 (14.2%)	1267 (31.7%)
273	AdMed_Hypotensive Agents	1	45 (0.3%)	45 (1.1%)
274	AdMed_Hypotensive Agents	NA	12168 (85.5%)	2684 (67.2%)
275	AdMed_Local Anesthetics	0	1820 (12.8%)	1254 (31.4%)
276	AdMed_Local Anesthetics	1	239 (1.7%)	58 (1.5%)
277	AdMed_Local Anesthetics	NA	12168 (85.5%)	2684 (67.2%)
278	AdMed_Miscellaneous Therapeutic Agents	0	1882 (13.2%)	1083 (27.1%)
279	AdMed_Miscellaneous Therapeutic Agents	1	177 (1.2%)	229 (5.7%)
280	AdMed_Miscellaneous Therapeutic Agents	NA	12168 (85.5%)	2684 (67.2%)
281	AdMed_Opiate Antagonists	0	1981 (13.9%)	1137 (28.5%)
282	AdMed_Opiate Antagonists	1	78 (0.5%)	175 (4.4%)
283	AdMed_Opiate Antagonists	NA	12168 (85.5%)	2684 (67.2%)
284	AdMed_OTHER	0	2059 (14.5%)	1311 (32.8%)
285	AdMed_OTHER	1	0 (0.0%)	1 (0.0%)
286	AdMed_OTHER	NA	12168 (85.5%)	2684 (67.2%)
287	AdMed_Oxytocics	0	2046 (14.4%)	1310 (32.8%)
288	AdMed_Oxytocics	1	13 (0.1%)	2 (0.1%)
289	AdMed_Oxytocics	NA	12168 (85.5%)	2684 (67.2%)
290	AdMed_Pharmaceutical Aids	0	2059 (14.5%)	1312 (32.8%)
291	AdMed_Pharmaceutical Aids	1	0 (0.0%)	0 (0.0%)
292	AdMed_Pharmaceutical Aids	NA	12168 (85.5%)	2684 (67.2%)
293	AdMed_Psychotherapeutic Agents	0	2029 (14.3%)	1252 (31.3%)
294	AdMed_Psychotherapeutic Agents	1	30 (0.2%)	60 (1.5%)

© 2018 Wong A et al. *JAMA Network Open.*

295	AdMed_Psychotherapeutic Agents	NA	12168 (85.5%)	2684 (67.2%)
296	AdMed_Renin-Angiotensin-Aldosterone System Inhibitors	0	2050 (14.4%)	1309 (32.8%)
297	AdMed_Renin-Angiotensin-Aldosterone System Inhibitors	1	9 (0.1%)	3 (0.1%)
298	AdMed_Renin-Angiotensin-Aldosterone System Inhibitors	NA	12168 (85.5%)	2684 (67.2%)
299	AdMed_Respiratory Tract Agents	0	1877 (13.2%)	1232 (30.8%)
300	AdMed_Respiratory Tract Agents	1	182 (1.3%)	80 (2.0%)
301	AdMed_Respiratory Tract Agents	NA	12168 (85.5%)	2684 (67.2%)
302	AdMed_Second Generation Antihistamines	0	2021 (14.2%)	1305 (32.7%)
303	AdMed_Second Generation Antihistamines	1	38 (0.3%)	7 (0.2%)
304	AdMed_Second Generation Antihistamines	NA	12168 (85.5%)	2684 (67.2%)
305	AdMed_Serums, Toxoids, and Vaccines	0	2057 (14.5%)	1296 (32.4%)
306	AdMed_Serums, Toxoids, and Vaccines	1	2 (0.0%)	16 (0.4%)
307	AdMed_Serums, Toxoids, and Vaccines	NA	12168 (85.5%)	2684 (67.2%)
308	AdMed_Skeletal Muscle Relaxants	0	2007 (14.1%)	1275 (31.9%)
309	AdMed_Skeletal Muscle Relaxants	1	52 (0.4%)	37 (0.9%)
310	AdMed_Skeletal Muscle Relaxants	NA	12168 (85.5%)	2684 (67.2%)
311	AdMed_Skin and Mucous Membrane Agents	0	2026 (14.2%)	1273 (31.9%)
312	AdMed_Skin and Mucous Membrane Agents	1	33 (0.2%)	39 (1.0%)
313	AdMed_Skin and Mucous Membrane Agents	NA	12168 (85.5%)	2684 (67.2%)
314	AdMed_Smooth Muscle Relaxants	0	2057 (14.5%)	1312 (32.8%)
315	AdMed_Smooth Muscle Relaxants	1	2 (0.0%)	0 (0.0%)
316	AdMed_Smooth Muscle Relaxants	NA	12168 (85.5%)	2684 (67.2%)
317	AdMed_Sympatholytic (Adrenergic Blocking) Agents	0	2031 (14.3%)	1278 (32.0%)
318	AdMed_Sympatholytic (Adrenergic Blocking) Agents	1	28 (0.2%)	34 (0.9%)
319	AdMed_Sympatholytic (Adrenergic Blocking)	NA	12168 (85.5%)	2684 (67.2%)

© 2018 Wong A et al. *JAMA Network Open.*

	Agents			
320	AdMed_Sympathomimetic (Adrenergic) Agents	0	1979 (13.9%)	1288 (32.2%)
321	AdMed_Sympathomimetic (Adrenergic) Agents	1	80 (0.6%)	24 (0.6%)
322	AdMed_Sympathomimetic (Adrenergic) Agents	NA	12168 (85.5%)	2684 (67.2%)
323	AdMed_Vasodilating Agents	0	2049 (14.4%)	1295 (32.4%)
324	AdMed_Vasodilating Agents	1	10 (0.1%)	17 (0.4%)
325	AdMed_Vasodilating Agents	NA	12168 (85.5%)	2684 (67.2%)
326	AdMed_Vitamins	0	1968 (13.8%)	1250 (31.3%)
327	AdMed_Vitamins	1	91 (0.6%)	62 (1.6%)
328	AdMed_Vitamins	NA	12168 (85.5%)	2684 (67.2%)
329	HomeMed_alpha-Adrenergic Blocking Agents	0	12344 (86.8%)	3390 (84.8%)
330	HomeMed_alpha-Adrenergic Blocking Agents	1	1061 (7.5%)	358 (9.0%)
331	HomeMed_alpha-Adrenergic Blocking Agents	NA	822 (5.8%)	248 (6.2%)
332	HomeMed_Analgesics and Antipyretics	0	2954 (20.8%)	824 (20.6%)
333	HomeMed_Analgesics and Antipyretics	1	10451 (73.5%)	2924 (73.2%)
334	HomeMed_Analgesics and Antipyretics	NA	822 (5.8%)	248 (6.2%)
335	HomeMed_Anorexigenic Agents and Respiratory and Cerebral Stimulants	0	12882 (90.5%)	3606 (90.2%)
336	HomeMed_Anorexigenic Agents and Respiratory and Cerebral Stimulants	1	523 (3.7%)	142 (3.6%)
337	HomeMed_Anorexigenic Agents and Respiratory and Cerebral Stimulants	NA	822 (5.8%)	248 (6.2%)
338	HomeMed_Anti-infective Agents	0	9744 (68.5%)	2724 (68.2%)
339	HomeMed_Anti-infective Agents	1	3661 (25.7%)	1024 (25.6%)
340	HomeMed_Anti-infective Agents	NA	822 (5.8%)	248 (6.2%)
341	HomeMed_Anticholinergic Agents	0	12331 (86.7%)	3420 (85.6%)
342	HomeMed_Anticholinergic Agents	1	1074 (7.5%)	328 (8.2%)
343	HomeMed_Anticholinergic Agents	NA	822 (5.8%)	248 (6.2%)
344	HomeMed_Anticonvulsant	0	8088 (56.8%)	2205 (55.2%)

© 2018 Wong A et al. *JAMA Network Open.*

	s			
345	HomeMed_Anticonvulsants	1	5317 (37.4%)	1543 (38.6%)
346	HomeMed_Anticonvulsants	NA	822 (5.8%)	248 (6.2%)
347	HomeMed_Antilipemic Agents	0	8588 (60.4%)	2266 (56.7%)
348	HomeMed_Antilipemic Agents	1	4817 (33.9%)	1482 (37.1%)
349	HomeMed_Antilipemic Agents	NA	822 (5.8%)	248 (6.2%)
350	HomeMed_Antimanic Agents	0	13216 (92.9%)	3708 (92.8%)
351	HomeMed_Antimanic Agents	1	189 (1.3%)	40 (1.0%)
352	HomeMed_Antimanic Agents	NA	822 (5.8%)	248 (6.2%)
353	HomeMed_Antimigraine Agents	0	9251 (65.0%)	2473 (61.9%)
354	HomeMed_Antimigraine Agents	1	4154 (29.2%)	1275 (31.9%)
355	HomeMed_Antimigraine Agents	NA	822 (5.8%)	248 (6.2%)
356	HomeMed_Antineoplastic Agents	0	12506 (87.9%)	3500 (87.6%)
357	HomeMed_Antineoplastic Agents	1	899 (6.3%)	248 (6.2%)
358	HomeMed_Antineoplastic Agents	NA	822 (5.8%)	248 (6.2%)
359	HomeMed_Antiparkinsonian Agents	0	13047 (91.7%)	3651 (91.4%)
360	HomeMed_Antiparkinsonian Agents	1	358 (2.5%)	97 (2.4%)
361	HomeMed_Antiparkinsonian Agents	NA	822 (5.8%)	248 (6.2%)
362	HomeMed_Anxiolytics, Sedatives, and Hypnotics	0	9331 (65.6%)	2673 (66.9%)
363	HomeMed_Anxiolytics, Sedatives, and Hypnotics	1	4074 (28.6%)	1075 (26.9%)
364	HomeMed_Anxiolytics, Sedatives, and Hypnotics	NA	822 (5.8%)	248 (6.2%)
365	HomeMed_Autonomic Drugs, Miscellaneous	0	13073 (91.9%)	3648 (91.3%)
366	HomeMed_Autonomic Drugs, Miscellaneous	1	332 (2.3%)	100 (2.5%)
367	HomeMed_Autonomic Drugs, Miscellaneous	NA	822 (5.8%)	248 (6.2%)
368	HomeMed_beta-Adrenergic Blocking Agents	0	9991 (70.2%)	2671 (66.8%)
369	HomeMed_beta-Adrenergic Blocking Agents	1	3414 (24.0%)	1077 (27.0%)
370	HomeMed_beta-	NA	822 (5.8%)	248 (6.2%)

© 2018 Wong A et al. *JAMA Network Open.*

	Adrenergic Blocking Agents			
371	HomeMed_Blood Derivatives	0	13401 (94.2%)	3748 (93.8%)
372	HomeMed_Blood Derivatives	1	4 (0.0%)	0 (0.0%)
373	HomeMed_Blood Derivatives	NA	822 (5.8%)	248 (6.2%)
374	HomeMed_Blood Formation, Coagulation, and Thrombosis	0	7414 (52.1%)	1930 (48.3%)
375	HomeMed_Blood Formation, Coagulation, and Thrombosis	1	5991 (42.1%)	1818 (45.5%)
376	HomeMed_Blood Formation, Coagulation, and Thrombosis	NA	822 (5.8%)	248 (6.2%)
377	HomeMed_Calcium-Channel Blocking Agents	0	11025 (77.5%)	3077 (77.0%)
378	HomeMed_Calcium-Channel Blocking Agents	1	2380 (16.7%)	671 (16.8%)
379	HomeMed_Calcium-Channel Blocking Agents	NA	822 (5.8%)	248 (6.2%)
380	HomeMed_Cardiac Drugs	0	9557 (67.2%)	2527 (63.2%)
381	HomeMed_Cardiac Drugs	1	3848 (27.0%)	1221 (30.6%)
382	HomeMed_Cardiac Drugs	NA	822 (5.8%)	248 (6.2%)
383	HomeMed_Cellular Therapy	0	13404 (94.2%)	3748 (93.8%)
384	HomeMed_Cellular Therapy	1	1 (0.0%)	0 (0.0%)
385	HomeMed_Cellular Therapy	NA	822 (5.8%)	248 (6.2%)
386	HomeMed_Central Nervous System Agents, Miscellaneous	0	13216 (92.9%)	3675 (92.0%)
387	HomeMed_Central Nervous System Agents, Miscellaneous	1	189 (1.3%)	73 (1.8%)
388	HomeMed_Central Nervous System Agents, Miscellaneous	NA	822 (5.8%)	248 (6.2%)
389	HomeMed_Dental Agents	0	13402 (94.2%)	3748 (93.8%)
390	HomeMed_Dental Agents	1	3 (0.0%)	0 (0.0%)
391	HomeMed_Dental Agents	NA	822 (5.8%)	248 (6.2%)
392	HomeMed_Devices	0	12020 (84.5%)	3308 (82.8%)
393	HomeMed_Devices	1	1385 (9.7%)	440 (11.0%)
394	HomeMed_Devices	NA	822 (5.8%)	248 (6.2%)
395	HomeMed_Diagnostic Agents	0	12667 (89.0%)	3480 (87.1%)
396	HomeMed_Diagnostic Agents	1	738 (5.2%)	268 (6.7%)

© 2018 Wong A et al. *JAMA Network Open.*

397	HomeMed_Diagnostic Agents	NA	822 (5.8%)	248 (6.2%)
398	HomeMed_Disinfectants (for agents used on objects other than skin)	0	13391 (94.1%)	3748 (93.8%)
399	HomeMed_Disinfectants (for agents used on objects other than skin)	1	14 (0.1%)	0 (0.0%)
400	HomeMed_Disinfectants (for agents used on objects other than skin)	NA	822 (5.8%)	248 (6.2%)
401	HomeMed_Electrolytic, Caloric, and Water Balance	0	7368 (51.8%)	1970 (49.3%)
402	HomeMed_Electrolytic, Caloric, and Water Balance	1	6037 (42.4%)	1778 (44.5%)
403	HomeMed_Electrolytic, Caloric, and Water Balance	NA	822 (5.8%)	248 (6.2%)
404	HomeMed_Enzymes	0	13317 (93.6%)	3730 (93.3%)
405	HomeMed_Enzymes	1	88 (0.6%)	18 (0.5%)
406	HomeMed_Enzymes	NA	822 (5.8%)	248 (6.2%)
407	HomeMed_Eye, Ear, Nose, and Throat (EENT) Preparations	0	10817 (76.0%)	2979 (74.5%)
408	HomeMed_Eye, Ear, Nose, and Throat (EENT) Preparations	1	2588 (18.2%)	769 (19.2%)
409	HomeMed_Eye, Ear, Nose, and Throat (EENT) Preparations	NA	822 (5.8%)	248 (6.2%)
410	HomeMed_Fibromyalgia Agents	0	12546 (88.2%)	3520 (88.1%)
411	HomeMed_Fibromyalgia Agents	1	859 (6.0%)	228 (5.7%)
412	HomeMed_Fibromyalgia Agents	NA	822 (5.8%)	248 (6.2%)
413	HomeMed_First Generation Antihistamines	0	11961 (84.1%)	3364 (84.2%)
414	HomeMed_First Generation Antihistamines	1	1444 (10.1%)	384 (9.6%)
415	HomeMed_First Generation Antihistamines	NA	822 (5.8%)	248 (6.2%)
416	HomeMed_Gastrointestinal Drugs	0	4950 (34.8%)	1317 (33.0%)
417	HomeMed_Gastrointestinal Drugs	1	8455 (59.4%)	2431 (60.8%)
418	HomeMed_Gastrointestinal Drugs	NA	822 (5.8%)	248 (6.2%)
419	HomeMed_General Anesthetics	0	13398 (94.2%)	3739 (93.6%)
420	HomeMed_General Anesthetics	1	7 (0.0%)	9 (0.2%)

© 2018 Wong A et al. *JAMA Network Open.*

421	HomeMed_General Anesthetics	NA	822 (5.8%)	248 (6.2%)
422	HomeMed_Heavy Metal Antagonists	0	13367 (94.0%)	3736 (93.5%)
423	HomeMed_Heavy Metal Antagonists	1	38 (0.3%)	12 (0.3%)
424	HomeMed_Heavy Metal Antagonists	NA	822 (5.8%)	248 (6.2%)
425	HomeMed_Hormones and Synthetic Substitutes	0	6990 (49.1%)	1930 (48.3%)
426	HomeMed_Hormones and Synthetic Substitutes	1	6415 (45.1%)	1818 (45.5%)
427	HomeMed_Hormones and Synthetic Substitutes	NA	822 (5.8%)	248 (6.2%)
428	HomeMed_Hypotensive Agents	0	6235 (43.8%)	1675 (41.9%)
429	HomeMed_Hypotensive Agents	1	7170 (50.4%)	2073 (51.9%)
430	HomeMed_Hypotensive Agents	NA	822 (5.8%)	248 (6.2%)
431	HomeMed_Local Anesthetics	0	13395 (94.2%)	3747 (93.8%)
432	HomeMed_Local Anesthetics	1	10 (0.1%)	1 (0.0%)
433	HomeMed_Local Anesthetics	NA	822 (5.8%)	248 (6.2%)
434	HomeMed_Miscellaneous Therapeutic Agents	0	8640 (60.7%)	2325 (58.2%)
435	HomeMed_Miscellaneous Therapeutic Agents	1	4765 (33.5%)	1423 (35.6%)
436	HomeMed_Miscellaneous Therapeutic Agents	NA	822 (5.8%)	248 (6.2%)
437	HomeMed_Opiate Antagonists	0	13241 (93.1%)	3680 (92.1%)
438	HomeMed_Opiate Antagonists	1	164 (1.2%)	68 (1.7%)
439	HomeMed_Opiate Antagonists	NA	822 (5.8%)	248 (6.2%)
440	HomeMed_OTHER	0	13361 (93.9%)	3736 (93.5%)
441	HomeMed_OTHER	1	44 (0.3%)	12 (0.3%)
442	HomeMed_OTHER	NA	822 (5.8%)	248 (6.2%)
443	HomeMed_Other Antihistamines	0	13405 (94.2%)	3748 (93.8%)
444	HomeMed_Other Antihistamines	1	0 (0.0%)	0 (0.0%)
445	HomeMed_Other Antihistamines	NA	822 (5.8%)	248 (6.2%)
446	HomeMed_Oxytocics	0	13405 (94.2%)	3748 (93.8%)
447	HomeMed_Oxytocics	1	0 (0.0%)	0 (0.0%)
448	HomeMed_Oxytocics	NA	822 (5.8%)	248 (6.2%)
449	HomeMed_Parasympathomimetic (Cholinergic)	0	13226 (93.0%)	3701 (92.6%)

© 2018 Wong A et al. *JAMA Network Open.*

	Agents			
450	HomeMed_Parasympathomimetic (Cholinergic) Agents	1	179 (1.3%)	47 (1.2%)
451	HomeMed_Parasympathomimetic (Cholinergic) Agents	NA	822 (5.8%)	248 (6.2%)
452	HomeMed_Pharmaceutical Aids	0	13122 (92.2%)	3660 (91.6%)
453	HomeMed_Pharmaceutical Aids	1	283 (2.0%)	88 (2.2%)
454	HomeMed_Pharmaceutical Aids	NA	822 (5.8%)	248 (6.2%)
455	HomeMed_Psychotherapeutic Agents	0	8657 (60.8%)	2412 (60.4%)
456	HomeMed_Psychotherapeutic Agents	1	4748 (33.4%)	1336 (33.4%)
457	HomeMed_Psychotherapeutic Agents	NA	822 (5.8%)	248 (6.2%)
458	HomeMed_Renin-Angiotensin-Aldosterone System Inhibitors	0	9258 (65.1%)	2576 (64.5%)
459	HomeMed_Renin-Angiotensin-Aldosterone System Inhibitors	1	4147 (29.1%)	1172 (29.3%)
460	HomeMed_Renin-Angiotensin-Aldosterone System Inhibitors	NA	822 (5.8%)	248 (6.2%)
461	HomeMed_Respiratory Tract Agents	0	8694 (61.1%)	2399 (60.0%)
462	HomeMed_Respiratory Tract Agents	1	4711 (33.1%)	1349 (33.8%)
463	HomeMed_Respiratory Tract Agents	NA	822 (5.8%)	248 (6.2%)
464	HomeMed_Second Generation Antihistamines	0	12038 (84.6%)	3375 (84.5%)
465	HomeMed_Second Generation Antihistamines	1	1367 (9.6%)	373 (9.3%)
466	HomeMed_Second Generation Antihistamines	NA	822 (5.8%)	248 (6.2%)
467	HomeMed_Serums, Toxoids, and Vaccines	0	13338 (93.8%)	3733 (93.4%)
468	HomeMed_Serums, Toxoids, and Vaccines	1	67 (0.5%)	15 (0.4%)
469	HomeMed_Serums, Toxoids, and Vaccines	NA	822 (5.8%)	248 (6.2%)
470	HomeMed_Skeletal Muscle Relaxants	0	11780 (82.8%)	3317 (83.0%)
471	HomeMed_Skeletal Muscle Relaxants	1	1625 (11.4%)	431 (10.8%)
472	HomeMed_Skeletal Muscle Relaxants	NA	822 (5.8%)	248 (6.2%)
473	HomeMed_Skin and Mucous Membrane Agents	0	10037 (70.5%)	2775 (69.4%)

© 2018 Wong A et al. *JAMA Network Open.*

474	HomeMed_Skin and Mucous Membrane Agents	1	3368 (23.7%)	973 (24.3%)
475	HomeMed_Skin and Mucous Membrane Agents	NA	822 (5.8%)	248 (6.2%)
476	HomeMed_Smooth Muscle Relaxants	0	12995 (91.3%)	3617 (90.5%)
477	HomeMed_Smooth Muscle Relaxants	1	410 (2.9%)	131 (3.3%)
478	HomeMed_Smooth Muscle Relaxants	NA	822 (5.8%)	248 (6.2%)
479	HomeMed_Sympatholytic (Adrenergic Blocking) Agents	0	9246 (65.0%)	2470 (61.8%)
480	HomeMed_Sympatholytic (Adrenergic Blocking) Agents	1	4159 (29.2%)	1278 (32.0%)
481	HomeMed_Sympatholytic (Adrenergic Blocking) Agents	NA	822 (5.8%)	248 (6.2%)
482	HomeMed_Sympathomimetic (Adrenergic) Agents	0	10551 (74.2%)	2876 (72.0%)
483	HomeMed_Sympathomimetic (Adrenergic) Agents	1	2854 (20.1%)	872 (21.8%)
484	HomeMed_Sympathomimetic (Adrenergic) Agents	NA	822 (5.8%)	248 (6.2%)
485	HomeMed_Vasodilating Agents	0	10397 (73.1%)	2846 (71.2%)
486	HomeMed_Vasodilating Agents	1	3008 (21.1%)	902 (22.6%)
487	HomeMed_Vasodilating Agents	NA	822 (5.8%)	248 (6.2%)
488	HomeMed_Vitamins	0	6111 (43.0%)	1651 (41.3%)
489	HomeMed_Vitamins	1	7294 (51.3%)	2097 (52.5%)
490	HomeMed_Vitamins	NA	822 (5.8%)	248 (6.2%)
491	Admit Category	Emergency	5512 (38.7%)	1714 (42.9%)
492	Admit Category	OTHER	6 (0.0%)	1 (0.0%)
493	Admit Category	Routine/Elective	6153 (43.2%)	1521 (38.1%)
494	Admit Category	Transplant/Donor	71 (0.5%)	11 (0.3%)
495	Admit Category	Urgent	2485 (17.5%)	749 (18.7%)
496	Admit Source	Physician Referral	6495 (45.7%)	1649 (41.3%)
497	Admit Source	Emergency Room	5432 (38.2%)	1615 (40.4%)
498	Admit Source	Transfer - Acute Hospital	921 (6.5%)	275 (6.9%)
499	Admit Source	OTHER	693 (4.9%)	216 (5.4%)
500	Admit Source	Clinic Referral	616 (4.3%)	206 (5.2%)
501	Admit Source	Self Referred	28 (0.2%)	20 (0.5%)
502	Admit Source	(Other)	42 (0.3%)	15 (0.4%)
503	Admit Service	Hospital Medicine	4019 (28.2%)	1264 (31.6%)
504	Admit Service	OTHER	2672 (18.8%)	801 (20.0%)

505	Admit Service	Orthopedics	2557 (18.0%)	558 (14.0%)
506	Admit Service	Neurosurgery	1842 (12.9%)	495 (12.4%)
507	Admit Service	General Surgery	1717 (12.1%)	378 (9.5%)
508	Admit Service	Neurology	774 (5.4%)	297 (7.4%)
509	Admit Service	(Other)	646 (4.5%)	203 (5.1%)
510	Admit Department	NEUR TRAN	250 (1.8%)	78 (2.0%)
511	Admit Department	NEUROSCIENCES 8L	1189 (8.4%)	266 (6.7%)
512	Admit Department	OTHER	7784 (54.7%)	2499 (62.5%)
513	Admit Department	PERIOP	5004 (35.2%)	1153 (28.9%)
514	Readmission within 30 days	No	12925 (90.8%)	3462 (86.6%)
515	Readmission within 30 days	Yes	1302 (9.2%)	534 (13.4%)
516	Readmission within 90 days	No	12931 (90.9%)	3436 (86.0%)
517	Readmission within 90 days	Yes	1296 (9.1%)	560 (14.0%)
518	CHF	No	12862 (90.4%)	3588 (89.8%)
519	CHF	Yes	347 (2.4%)	145 (3.6%)
520	CHF	NA	1018 (7.2%)	263 (6.6%)
521	Arrhythmia	No	12664 (89.0%)	3539 (88.6%)
522	Arrhythmia	Yes	545 (3.8%)	194 (4.9%)
523	Arrhythmia	NA	1018 (7.2%)	263 (6.6%)
524	Valvular	No	13074 (91.9%)	3701 (92.6%)
525	Valvular	Yes	135 (0.9%)	32 (0.8%)
526	Valvular	NA	1018 (7.2%)	263 (6.6%)
527	PHTN	No	13021 (91.5%)	3672 (91.9%)
528	PHTN	Yes	188 (1.3%)	61 (1.5%)
529	PHTN	NA	1018 (7.2%)	263 (6.6%)
530	PVD	No	12923 (90.8%)	3656 (91.5%)
531	PVD	Yes	286 (2.0%)	77 (1.9%)
532	PVD	NA	1018 (7.2%)	263 (6.6%)
533	HTN	No	12534 (88.1%)	3605 (90.2%)
534	HTN	Yes	675 (4.7%)	128 (3.2%)
535	HTN	NA	1018 (7.2%)	263 (6.6%)
536	Paralysis	No	13146 (92.4%)	3725 (93.2%)
537	Paralysis	Yes	63 (0.4%)	8 (0.2%)
538	Paralysis	NA	1018 (7.2%)	263 (6.6%)
539	NeuroOther	No	12735 (89.5%)	3592 (89.9%)
540	NeuroOther	Yes	474 (3.3%)	141 (3.5%)
541	NeuroOther	NA	1018 (7.2%)	263 (6.6%)

© 2018 Wong A et al. *JAMA Network Open.*

542	Pulmonary	No	12832 (90.2%)	3628 (90.8%)
543	Pulmonary	Yes	377 (2.6%)	105 (2.6%)
544	Pulmonary	NA	1018 (7.2%)	263 (6.6%)
545	DM	No	12801 (90.0%)	3657 (91.5%)
546	DM	Yes	408 (2.9%)	76 (1.9%)
547	DM	NA	1018 (7.2%)	263 (6.6%)
548	DMcx	No	13114 (92.2%)	3707 (92.8%)
549	DMcx	Yes	95 (0.7%)	26 (0.7%)
550	DMcx	NA	1018 (7.2%)	263 (6.6%)
551	Hypothyroid	No	13112 (92.2%)	3706 (92.7%)
552	Hypothyroid	Yes	97 (0.7%)	27 (0.7%)
553	Hypothyroid	NA	1018 (7.2%)	263 (6.6%)
554	Renal	No	12773 (89.8%)	3617 (90.5%)
555	Renal	Yes	436 (3.1%)	116 (2.9%)
556	Renal	NA	1018 (7.2%)	263 (6.6%)
557	Liver	No	12817 (90.1%)	3625 (90.7%)
558	Liver	Yes	392 (2.8%)	108 (2.7%)
559	Liver	NA	1018 (7.2%)	263 (6.6%)
560	PUD	No	13196 (92.8%)	3727 (93.3%)
561	PUD	Yes	13 (0.1%)	6 (0.2%)
562	PUD	NA	1018 (7.2%)	263 (6.6%)
563	HIV	No	13142 (92.4%)	3716 (93.0%)
564	HIV	Yes	67 (0.5%)	17 (0.4%)
565	HIV	NA	1018 (7.2%)	263 (6.6%)
566	Lymphoma	No	12862 (90.4%)	3670 (91.8%)
567	Lymphoma	Yes	347 (2.4%)	63 (1.6%)
568	Lymphoma	NA	1018 (7.2%)	263 (6.6%)
569	Mets	No	12944 (91.0%)	3631 (90.9%)
570	Mets	Yes	265 (1.9%)	102 (2.6%)
571	Mets	NA	1018 (7.2%)	263 (6.6%)
572	Tumor	No	12247 (86.1%)	3430 (85.8%)
573	Tumor	Yes	962 (6.8%)	303 (7.6%)
574	Tumor	NA	1018 (7.2%)	263 (6.6%)
575	Rheumatic	No	13063 (91.8%)	3699 (92.6%)
576	Rheumatic	Yes	146 (1.0%)	34 (0.9%)
577	Rheumatic	NA	1018 (7.2%)	263 (6.6%)
578	Coagulopathy	No	13077 (91.9%)	3700 (92.6%)
579	Coagulopathy	Yes	132 (0.9%)	33 (0.8%)
580	Coagulopathy	NA	1018 (7.2%)	263 (6.6%)

© 2018 Wong A et al. *JAMA Network Open.*

581	Obesity	No	13042 (91.7%)	3694 (92.4%)
582	Obesity	Yes	167 (1.2%)	39 (1.0%)
583	Obesity	NA	1018 (7.2%)	263 (6.6%)
584	WeightLoss	No	13092 (92.0%)	3707 (92.8%)
585	WeightLoss	Yes	117 (0.8%)	26 (0.7%)
586	WeightLoss	NA	1018 (7.2%)	263 (6.6%)
587	FluidsLytes	No	12655 (89.0%)	3541 (88.6%)
588	FluidsLytes	Yes	554 (3.9%)	192 (4.8%)
589	FluidsLytes	NA	1018 (7.2%)	263 (6.6%)
590	BloodLoss	No	13195 (92.7%)	3729 (93.3%)
591	BloodLoss	Yes	14 (0.1%)	4 (0.1%)
592	BloodLoss	NA	1018 (7.2%)	263 (6.6%)
593	Anemia	No	13178 (92.6%)	3725 (93.2%)
594	Anemia	Yes	31 (0.2%)	8 (0.2%)
595	Anemia	NA	1018 (7.2%)	263 (6.6%)
596	Alcohol	No	13105 (92.1%)	3696 (92.5%)
597	Alcohol	Yes	104 (0.7%)	37 (0.9%)
598	Alcohol	NA	1018 (7.2%)	263 (6.6%)
599	Drugs	No	13104 (92.1%)	3716 (93.0%)
600	Drugs	Yes	105 (0.7%)	17 (0.4%)
601	Drugs	NA	1018 (7.2%)	263 (6.6%)
602	Psychoses	No	13209 (92.8%)	3733 (93.4%)
603	Psychoses	Yes	0 (0.0%)	0 (0.0%)
604	Psychoses	NA	1018 (7.2%)	263 (6.6%)
605	Depression	No	13058 (91.8%)	3707 (92.8%)
606	Depression	Yes	151 (1.1%)	26 (0.7%)
607	Depression	NA	1018 (7.2%)	263 (6.6%)
608	O2 Device_Aerosol mask	0	13251 (93.1%)	3751 (93.9%)
609	O2 Device_Aerosol mask	1	96 (0.7%)	24 (0.6%)
610	O2 Device_Aerosol mask	NA	880 (6.2%)	221 (5.5%)
611	O2 Device_BiPAP	0	13206 (92.8%)	3742 (93.6%)
612	O2 Device_BiPAP	1	141 (1.0%)	33 (0.8%)
613	O2 Device_BiPAP	NA	880 (6.2%)	221 (5.5%)
614	O2 Device_Blow-by	0	13323 (93.6%)	3769 (94.3%)
615	O2 Device_Blow-by	1	24 (0.2%)	6 (0.2%)
616	O2 Device_Blow-by	NA	880 (6.2%)	221 (5.5%)
617	O2 Device_CPAP	0	12977 (91.2%)	3652 (91.4%)
618	O2 Device_CPAP	1	370 (2.6%)	123 (3.1%)
619	O2 Device_CPAP	NA	880 (6.2%)	221 (5.5%)

© 2018 Wong A et al. *JAMA Network Open.*

620	O2 Device_Face tent	0	13182 (92.7%)	3737 (93.5%)
621	O2 Device_Face tent	1	165 (1.2%)	38 (1.0%)
622	O2 Device_Face tent	NA	880 (6.2%)	221 (5.5%)
623	O2 Device_High flow nasal cannula	0	13222 (92.9%)	3740 (93.6%)
624	O2 Device_High flow nasal cannula	1	125 (0.9%)	35 (0.9%)
625	O2 Device_High flow nasal cannula	NA	880 (6.2%)	221 (5.5%)
626	O2 Device_Nasal cannula	0	6237 (43.8%)	1876 (46.9%)
627	O2 Device_Nasal cannula	1	7110 (50.0%)	1899 (47.5%)
628	O2 Device_Nasal cannula	NA	880 (6.2%)	221 (5.5%)
629	O2 Device_Non-rebreather mask	0	13336 (93.7%)	3772 (94.4%)
630	O2 Device_Non-rebreather mask	1	11 (0.1%)	3 (0.1%)
631	O2 Device_Non-rebreather mask	NA	880 (6.2%)	221 (5.5%)
632	O2 Device_None (Room air)	0	8034 (56.5%)	2157 (54.0%)
633	O2 Device_None (Room air)	1	5313 (37.3%)	1618 (40.5%)
634	O2 Device_None (Room air)	NA	880 (6.2%)	221 (5.5%)
635	O2 Device_Other (Comment)	0	13345 (93.8%)	3775 (94.5%)
636	O2 Device_Other (Comment)	1	2 (0.0%)	0 (0.0%)
637	O2 Device_Other (Comment)	NA	880 (6.2%)	221 (5.5%)
638	O2 Device_Simple mask	0	13347 (93.8%)	3775 (94.5%)
639	O2 Device_Simple mask	1	0 (0.0%)	0 (0.0%)
640	O2 Device_Simple mask	NA	880 (6.2%)	221 (5.5%)
641	O2 Device_T-Piece	0	13346 (93.8%)	3775 (94.5%)
642	O2 Device_T-Piece	1	1 (0.0%)	0 (0.0%)
643	O2 Device_T-Piece	NA	880 (6.2%)	221 (5.5%)
644	O2 Device_Trach mask	0	13343 (93.8%)	3775 (94.5%)
645	O2 Device_Trach mask	1	4 (0.0%)	0 (0.0%)
646	O2 Device_Trach mask	NA	880 (6.2%)	221 (5.5%)
647	*Braces/Devices/Sensory Aids_Abdominal binder	0	12881 (90.5%)	3662 (91.6%)
648	*Braces/Devices/Sensory Aids_Abdominal binder	1	445 (3.1%)	107 (2.7%)
649	*Braces/Devices/Sensory Aids_Abdominal binder	NA	901 (6.3%)	227 (5.7%)
650	*Braces/Devices/Sensory Aids_Abductor pillow	0	13226 (93.0%)	3738 (93.5%)
651	*Braces/Devices/Sensory Aids_Abductor pillow	1	100 (0.7%)	31 (0.8%)

© 2018 Wong A et al. *JAMA Network Open.*

652	*Braces/Devices/Sensory Aids_Abductor pillow	NA	901 (6.3%)	227 (5.7%)
653	*Braces/Devices/Sensory Aids_Brace	0	12523 (88.0%)	3593 (89.9%)
654	*Braces/Devices/Sensory Aids_Brace	1	803 (5.6%)	176 (4.4%)
655	*Braces/Devices/Sensory Aids_Brace	NA	901 (6.3%)	227 (5.7%)
656	*Braces/Devices/Sensory Aids_Chest binder	0	13325 (93.7%)	3768 (94.3%)
657	*Braces/Devices/Sensory Aids_Chest binder	1	1 (0.0%)	1 (0.0%)
658	*Braces/Devices/Sensory Aids_Chest binder	NA	901 (6.3%)	227 (5.7%)
659	*Braces/Devices/Sensory Aids_Collar	0	12952 (91.0%)	3662 (91.6%)
660	*Braces/Devices/Sensory Aids_Collar	1	374 (2.6%)	107 (2.7%)
661	*Braces/Devices/Sensory Aids_Collar	NA	901 (6.3%)	227 (5.7%)
662	*Braces/Devices/Sensory Aids_External fixator	0	13286 (93.4%)	3769 (94.3%)
663	*Braces/Devices/Sensory Aids_External fixator	1	40 (0.3%)	0 (0.0%)
664	*Braces/Devices/Sensory Aids_External fixator	NA	901 (6.3%)	227 (5.7%)
665	*Braces/Devices/Sensory Aids_Halo	0	13325 (93.7%)	3769 (94.3%)
666	*Braces/Devices/Sensory Aids_Halo	1	1 (0.0%)	0 (0.0%)
667	*Braces/Devices/Sensory Aids_Halo	NA	901 (6.3%)	227 (5.7%)
668	*Braces/Devices/Sensory Aids_Helmet	0	13308 (93.5%)	3761 (94.1%)
669	*Braces/Devices/Sensory Aids_Helmet	1	18 (0.1%)	8 (0.2%)
670	*Braces/Devices/Sensory Aids_Helmet	NA	901 (6.3%)	227 (5.7%)
671	*Braces/Devices/Sensory Aids_Immobilizer	0	13034 (91.6%)	3695 (92.5%)
672	*Braces/Devices/Sensory Aids_Immobilizer	1	292 (2.1%)	74 (1.9%)
673	*Braces/Devices/Sensory Aids_Immobilizer	NA	901 (6.3%)	227 (5.7%)
674	*Braces/Devices/Sensory Aids_None	0	3830 (26.9%)	1092 (27.3%)
675	*Braces/Devices/Sensory Aids_None	1	9496 (66.7%)	2677 (67.0%)
676	*Braces/Devices/Sensory Aids_None	NA	901 (6.3%)	227 (5.7%)
677	*Braces/Devices/Sensory Aids_Orthotics	0	13290 (93.4%)	3758 (94.0%)
678	*Braces/Devices/Sensory Aids_Orthotics	1	36 (0.3%)	11 (0.3%)

© 2018 Wong A et al. *JAMA Network Open.*

679	*Braces/Devices/Sensory Aids_Orthotics	NA	901 (6.3%)	227 (5.7%)
680	*Braces/Devices/Sensory Aids_Other (Comment)	0	13191 (92.7%)	3742 (93.6%)
681	*Braces/Devices/Sensory Aids_Other (Comment)	1	135 (0.9%)	27 (0.7%)
682	*Braces/Devices/Sensory Aids_Other (Comment)	NA	901 (6.3%)	227 (5.7%)
683	*Braces/Devices/Sensory Aids_Pavlik Harness	0	13324 (93.7%)	3769 (94.3%)
684	*Braces/Devices/Sensory Aids_Pavlik Harness	1	2 (0.0%)	0 (0.0%)
685	*Braces/Devices/Sensory Aids_Pavlik Harness	NA	901 (6.3%)	227 (5.7%)
686	*Braces/Devices/Sensory Aids_Prosthesis	0	13305 (93.5%)	3765 (94.2%)
687	*Braces/Devices/Sensory Aids_Prosthesis	1	21 (0.1%)	4 (0.1%)
688	*Braces/Devices/Sensory Aids_Prosthesis	NA	901 (6.3%)	227 (5.7%)
689	*Braces/Devices/Sensory Aids_Sensory aid(s)	0	11818 (83.1%)	3236 (81.0%)
690	*Braces/Devices/Sensory Aids_Sensory aid(s)	1	1508 (10.6%)	533 (13.3%)
691	*Braces/Devices/Sensory Aids_Sensory aid(s)	NA	901 (6.3%)	227 (5.7%)
692	*Braces/Devices/Sensory Aids_Sling	0	13232 (93.0%)	3755 (94.0%)
693	*Braces/Devices/Sensory Aids_Sling	1	94 (0.7%)	14 (0.4%)
694	*Braces/Devices/Sensory Aids_Sling	NA	901 (6.3%)	227 (5.7%)
695	*Braces/Devices/Sensory Aids_Splint	0	13217 (92.9%)	3748 (93.8%)
696	*Braces/Devices/Sensory Aids_Splint	1	109 (0.8%)	21 (0.5%)
697	*Braces/Devices/Sensory Aids_Splint	NA	901 (6.3%)	227 (5.7%)
698	Sleep Habit Details_Ear Plugs	0	1035 (7.3%)	262 (6.6%)
699	Sleep Habit Details_Ear Plugs	1	25 (0.2%)	7 (0.2%)
700	Sleep Habit Details_Ear Plugs	NA	13167 (92.5%)	3727 (93.3%)
701	Sleep Habit Details_Eye Mask	0	1045 (7.3%)	262 (6.6%)
702	Sleep Habit Details_Eye Mask	1	15 (0.1%)	7 (0.2%)
703	Sleep Habit Details_Eye Mask	NA	13167 (92.5%)	3727 (93.3%)
704	Sleep Habit Details_Fan	0	1031 (7.2%)	260 (6.5%)
705	Sleep Habit Details_Fan	1	29 (0.2%)	9 (0.2%)
706	Sleep Habit Details_Fan	NA	13167 (92.5%)	3727 (93.3%)

© 2018 Wong A et al. *JAMA Network Open.*

707	Sleep Habit Details_Lights Off	0	998 (7.0%)	248 (6.2%)
708	Sleep Habit Details_Lights Off	1	62 (0.4%)	21 (0.5%)
709	Sleep Habit Details_Lights Off	NA	13167 (92.5%)	3727 (93.3%)
710	Sleep Habit Details_Lights On	0	1047 (7.4%)	267 (6.7%)
711	Sleep Habit Details_Lights On	1	13 (0.1%)	2 (0.1%)
712	Sleep Habit Details_Lights On	NA	13167 (92.5%)	3727 (93.3%)
713	Sleep Habit Details_Music	0	1015 (7.1%)	266 (6.7%)
714	Sleep Habit Details_Music	1	45 (0.3%)	3 (0.1%)
715	Sleep Habit Details_Music	NA	13167 (92.5%)	3727 (93.3%)
716	Sleep Habit Details_Other (Comment)	0	937 (6.6%)	226 (5.7%)
717	Sleep Habit Details_Other (Comment)	1	123 (0.9%)	43 (1.1%)
718	Sleep Habit Details_Other (Comment)	NA	13167 (92.5%)	3727 (93.3%)
719	Sleep Habit Details_Sleep Medicine	0	288 (2.0%)	90 (2.3%)
720	Sleep Habit Details_Sleep Medicine	1	772 (5.4%)	179 (4.5%)
721	Sleep Habit Details_Sleep Medicine	NA	13167 (92.5%)	3727 (93.3%)
722	Sleep Habit Details_TV	0	990 (7.0%)	248 (6.2%)
723	Sleep Habit Details_TV	1	70 (0.5%)	21 (0.5%)
724	Sleep Habit Details_TV	NA	13167 (92.5%)	3727 (93.3%)
725	Relieving Factors_Cold	0	5193 (36.5%)	1314 (32.9%)
726	Relieving Factors_Cold	1	162 (1.1%)	40 (1.0%)
727	Relieving Factors_Cold	NA	8872 (62.4%)	2642 (66.1%)
728	Relieving Factors_Distractions	0	5177 (36.4%)	1303 (32.6%)
729	Relieving Factors_Distractions	1	178 (1.3%)	51 (1.3%)
730	Relieving Factors_Distractions	NA	8872 (62.4%)	2642 (66.1%)
731	Relieving Factors_Heat	0	5182 (36.4%)	1286 (32.2%)
732	Relieving Factors_Heat	1	173 (1.2%)	68 (1.7%)
733	Relieving Factors_Heat	NA	8872 (62.4%)	2642 (66.1%)
734	Relieving Factors_Massage	0	5313 (37.3%)	1347 (33.7%)
735	Relieving Factors_Massage	1	42 (0.3%)	7 (0.2%)
736	Relieving Factors_Massage	NA	8872 (62.4%)	2642 (66.1%)
737	Relieving Factors_Medication	0	953 (6.7%)	257 (6.4%)

© 2018 Wong A et al. *JAMA Network Open.*

738	Relieving Factors_Medication	1	4402 (30.9%)	1097 (27.5%)
739	Relieving Factors_Medication	NA	8872 (62.4%)	2642 (66.1%)
740	Relieving Factors_None	0	4776 (33.6%)	1188 (29.7%)
741	Relieving Factors_None	1	579 (4.1%)	166 (4.2%)
742	Relieving Factors_None	NA	8872 (62.4%)	2642 (66.1%)
743	Relieving Factors_Other (Comment)	0	5236 (36.8%)	1322 (33.1%)
744	Relieving Factors_Other (Comment)	1	119 (0.8%)	32 (0.8%)
745	Relieving Factors_Other (Comment)	NA	8872 (62.4%)	2642 (66.1%)
746	Relieving Factors_Relaxation	0	4692 (33.0%)	1192 (29.8%)
747	Relieving Factors_Relaxation	1	663 (4.7%)	162 (4.1%)
748	Relieving Factors_Relaxation	NA	8872 (62.4%)	2642 (66.1%)
749	Relieving Factors_Reposition	0	4625 (32.5%)	1165 (29.2%)
750	Relieving Factors_Reposition	1	730 (5.1%)	189 (4.7%)
751	Relieving Factors_Reposition	NA	8872 (62.4%)	2642 (66.1%)
752	Relieving Factors_Spiritual Care	0	5348 (37.6%)	1353 (33.9%)
753	Relieving Factors_Spiritual Care	1	7 (0.0%)	1 (0.0%)
754	Relieving Factors_Spiritual Care	NA	8872 (62.4%)	2642 (66.1%)

© 2018 Wong A et al. *JAMA Network Open.*

eTable 3. Confusion Matrix Metrics for tested delirium prediction models. PPV, positive predictive value; NPV, negative predictive value; NNS, number needed to screen; AWOL, validated delirium risk screening tool based on Age, ability to spell WORLD backwards, Orientation, and iLlness severity.

Binary Classification Model	Sensitivity (95% CI)	Specificity (95% CI)	PPV (95% CI)	NPV (95% CI)	NNS
**Gradient Boosting Machine** 90% specificity threshold (set at 7.1% probability of delirium)	59.7% (52.4-66.7%)	90.0% (89.0-90.9%)	23.1% (20.5-25.9%)	97.8% (97.4-98.1%)	4.8
**Gradient Boosting Machine** 90% sensitivity threshold (set at 1.9% probability of delirium)	90.0% (84.9-93.9%)	56.6% (55.0-58.2%)	9.4% (8.9-10.0%)	99.1% (98.7-99.4%)	12
**Penalized Logistic Regression** 90% specificity threshold (set at 7.5% probability of delirium)	56.5% (49.2-63.7%)	90.0% (89.0-91.0%)	22.1% (19.6-25.0%)	97.6% (97.2-98.0%)	5.1
**Penalized Logistic Regression** 90% sensitivity threshold (set at 2.3% probability of delirium)	90.0% (84.9-93.9%)	58.0% (56.4-59.5%)	9.7% (9.2-10.3%)	99.2% (98.7-99.4%)	11.3
**Random Forest** 90% specificity threshold (set at 11.5% probability of delirium)	59.2% (51.8-66.2%)	90.0% (89.0-91.0%)	22.9% (20.4-25.7%)	97.8% (97.4-98.1%)	4.8
**Random Forest** 90% sensitivity threshold (set at 3.6% probability of delirium)	90.0% (84.9-93.9%)	56.0% (54.4-57.6%)	9.3% (8.8-9.8%)	99.1% (98.7-99.4%)	11.8
**AWOL≥2**	32.8% (24.7-41.8%)	90.5% (89.5-91.5%)	11.8% (9.3-15.0%)	97.2% (96.9-97.5%)	11.1

© 2018 Wong A et al. *JAMA Network Open.*

eTable 4. Confusion Matrix for Gradient Boosting Machine Using 90% Specificity Threshold

	Reference	
Prediction	Positive Delirium Screen	Negative Delirium Screen
Delirium Predicted	114	380
Delirium Not Predicted	77	3425

© 2018 Wong A et al. *JAMA Network Open.*

eTable 5. Confusion Matrix for Gradient Boosting Machine Using 90% Sensitivity Threshold

	Reference	
Prediction	Positive Delirium Screen	Negative Delirium Screen
Delirium Predicted	172	1651
Delirium Not Predicted	19	2154

© 2018 Wong A et al. *JAMA Network Open.*

eTable 6. Confusion Matrix for Penalized Logistic Regression Using 90% Specificity Threshold

	Reference	
Prediction	Positive Delirium Screen	Negative Delirium Screen
Delirium Predicted	108	380
Delirium Not Predicted	83	3425

© 2018 Wong A et al. *JAMA Network Open.*

eTable 7. Confusion Matrix for Penalized Logistic Regression Using 90% Sensitivity Threshold

	Reference	
Prediction	Positive Delirium Screen	Negative Delirium Screen
Delirium Predicted	172	1600
Delirium Not Predicted	19	2205

© 2018 Wong A et al. *JAMA Network Open.*

eTable 8. Confusion Matrix for Random Forest Using 90% Specificity Threshold

	Reference	
Prediction	Positive Delirium Screen	Negative Delirium Screen
Delirium Predicted	113	380
Delirium Not Predicted	78	3425

© 2018 Wong A et al. *JAMA Network Open.*

eTable 9. Confusion Matrix for Random Forest Using 90% Sensitivity Threshold

	Reference	
Prediction	Positive Delirium Screen	Negative Delirium Screen
Delirium Predicted	172	1673
Delirium Not Predicted	19	2132

© 2018 Wong A et al. *JAMA Network Open.*

eTable 10. Confusion Matrix for AWOL Using AWOL≥2 Threshold. AWOL, validated delirium risk screening tool based on Age, ability to spell WORLD backwards, Orientation, and iLlness severity.

	Reference	
Prediction	Positive Delirium Screen	Negative Delirium Screen
Delirium Predicted	41	306
Delirium Not Predicted	84	2925

© 2018 Wong A et al. *JAMA Network Open.*
